# Utilizing Photothermal Effect Enhances Photocatalytic Water Splitting Coupled with Selective Benzyl Alcohol Oxidation over Schottky Junctions

**DOI:** 10.1002/advs.202501931

**Published:** 2025-04-11

**Authors:** Bojing Sun, Mengjia Ye, Yachao Xu, Ying Jiang, Dongfang Hou, Xiu‐qing Qiao, Meidi Wang, Yunchen Du, Dong‐Sheng Li

**Affiliations:** ^1^ College of Materials and Chemical Engineering Key Laboratory of Inorganic Nonmetallic Crystalline and Energy Conversion Materials China Three Gorges University Yichang Hubei 443002 P. R. China; ^2^ MIIT Key Laboratory of Critical Materials Technology for New Energy Conversion and Storage School of Chemistry and Chemical Engineering Harbin Institute of Technology Harbin 150001 China; ^3^ Hubei Three Gorges Laboratory Yichang Hubei 443007 P. R. China; ^4^ School of Materials Science and Engineering Peking University Beijing 100871 China

**Keywords:** defective ZnIn_2_S_4_, photocatalytic BA oxidation, photocatalytic water splitting, photothermal effect, Schottky junctions

## Abstract

As is well known, there are problems such as low utilization rate of photogenerated holes and resource consumption of sacrificial agent in solar‐driven photocatalytic water splitting to hydrogen technology. Herein, WC quantum dots decorated defective ZnIn_2_S_4_ nanosheets (DZIS/WCQDs) dual‐functional photocatalysts are fabricated. Its unique Schottky junctions and photothermal effect significantly promote the separation and transport efficiency of photogenerated carriers, as well as achieving synergistic enhancement of photocatalytic water splitting coupled with selective oxidation of benzyl alcohol (BA). Moreover, the photothermal effect can slowly induce the decomposition of H_2_O_2_ to produce ·OH, and the low concentration ·OH and photogenerated holes continuously generated in situ can directly and rapidly attack the αC─H bond of BA to improve benzaldehyde (BAD) conversion rate and selectivity. Consequently, DZIS/WCQDs composites exhibit a surprising conversion rate and selectivity of 85.34% and 96.53% for BAD, and outstanding H_2_ and BAD evolution rates of 12.58 and 10.53 mmol g^−1^ h^−1^ without sacrificial agent and co‐catalyst. Notably, combining the production rate and selectivity of products, the DZIS/WCQDs is the optimal catalyst material at present. This work opens up a green and carbon free effective path to solve the problems of low efficiency and high cost of photocatalytic hydrogen production.

## Graphical abstract

This study introduces a WC quantum dot decorated defective ZnIn_2_S_4_ nanosheets (DZIS/WCQDs) photocatalyst, which achieves synergistic enhancement of photocatalytic water splitting coupled with benzyl alcohol oxidation. It is demonstrated that the unique Schottky junctions and photothermal effect significantly promote the separation and transport of photogenerated carriers. The low concentration ·OH and photogenerated holes further improve benzaldehyde conversion rate and selectivity.

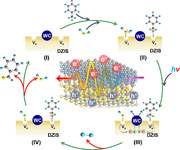

## 1 Introduction

Hydrogen is considered to be one of the ideal energy carrier to solve energy crisis and environmental pollution.^[^ ^1^ ^]^ The solar driven photocatalytic water splitting hydrogen production (2H_2_O → 2H_2_+O_2_) is known as the green holy grail technology and has attracted much attention from academia and industry.^[^ ^2^ ^]^ However, the oxygen evolution half reaction of photocatalytic water splitting has disadvantages such as high activation energy, slow reaction kinetics, low reaction value, and difficulty in separating the generated hydrogen and oxygen, which seriously restrict the entire photocatalytic process.^[^ ^3^ ^]^ In the past few decades, photocatalytic water splitting for hydrogen production has mainly focused on the research of photocatalytic partial water splitting for hydrogen production technology with high hydrogen production rate. Unfortunately, the catalytic process of partial water splitting requires continuous supplementation of sacrificial agents to consume photogenerated holes, resulting in increased costs and the production of by‐products (such as CO_2_), which is not conducive to practical industrial applications.^[^ ^4^ ^]^ Therefore, how to effectively consume and utilize photogenerated holes, and explore carbon free green pathways for photocatalytic hydrogen production, remains an urgent frontier research area that needs to be broken through.

Photocatalytic water splitting for hydrogen production coupled with selective organic oxidation using semiconductor‐based catalysts is one of the effective strategies to achieve efficient utilization of photogenerated holes and carbon free properties of photocatalytic hydrogen production.^[^ ^5^ ^]^ In the field of fine organic chemicals, benzyl alcohol (BA) is one of the simplest and most basic alcohol chemicals, and its oxidized benzaldehyde (BAD) is an important intermediate for the synthesis of fine chemicals, drugs, and pesticides.^[^ ^6^ ^]^ In traditional processes, BAD is produced from toluene through complex processes such as continuous photochlorination, amination, and acid hydrolysis, which are accompanied by a large amount of pollutants and wastewater.^[^ ^7^ ^]^ Undoubtedly, the photocatalytic selective oxidation of BA provides an environmentally friendly pathway for the production of BAD.

Normally, photocatalytic conversion of BA is driven by various reactive oxygen species (·O^2−^/H_2_O_2_/·OH/^1^O_2_) generated from oxygen/water and photogenerated holes as oxidants.^[^ ^8^ ^]^ It is worth noting that in the photocatalytic oxidation of BA, in order to avoid the generation of amount strong oxidizing ·OH by the catalyst oxidizing water, researchers usually use organic solvents such as acetonitrile or phenyl trifluoride as reaction media to reduce oxygen and generate mild oxidizing ·O^2−^ for the oxidation of BA to improve the selectivity of alcohol to aldehyde conversion.^[^ ^9^ ^]^ However, the potential environmental hazards and additional costs of organic solvents greatly limit the practical applications. Therefore, developing suitable photocatalysts using water as the reaction medium to achieve the coupling of water splitting and BA selective oxidation without oxygen and sacrificial agents is a reasonable and feasible catalytic strategy.

Bimetallic sulfides with narrow bandgap not only can absorb and utilize sunlight in the visible and near‐infrared regions, but also its suitable valence band positions effectively prevent the further oxidation of BAD in water.^[^ ^10^ ^]^ In addition, 2D layered bimetallic sulfides have the characteristics of adjustable electronic and energy level structure,^[^ ^11^ ^]^ designable non stoichiometric defect sites and surface state, and thus exhibiting great potential in water splitting hydrogen production coupled with selective oxidation of BA. For instance, Liu et al. successfully fabricated O‐ZnIn_2_S_4_/TiO_2‐_ *_x_* S‐scheme heterojunction through oxygen defect and doping engineering, and demonstrated that S‐scheme energy band structure alignment with higher redox potentials and larger Fermi level potential difference was conducive to interface charge transfer and separation.^[^ ^12^ ^]^ Moreover, Luo et al. constructed WO_3_/ZnIn_2_S_4_ hierarchical Z‐scheme composites, which could effectively separate photogenerated electrons and holes through the built‐in electric field, while retaining the strong reduction and oxidation capacity of the catalyst, and thus showing excellent photocatalytic water reduction and BA oxidation activities.^[^ ^13^ ^]^ Although S‐scheme or Z‐scheme heterojunction achieves a strong interfacial electric field, the energy band structure determines its high reduction and oxidation capacity. The strong oxidation capacity of catalysts is easy to over‐oxidize BA, which is not conducive to the selectivity of product. In addition, the fully occupied d‐orbitals are the essential features of Zn and In atoms (3d^10^), and hence the 2p orbitals of C or O atoms in BA usually form weak bons with the d orbitals of Zn and In atoms. This weak interaction makes the efficiency of photocatalytic BA oxidation reaction still unsatisfactory for ZnIn_2_S_4_. Therefore, it is necessary to design and develop a system with excellent photocatalytic active of water splitting coupled with BA oxidation, while enhancing the selectivity of BA oxidation through mild oxidation ability.

Herein, we design and synthesize WC quantum dots (WC QDs) modified defective ZnIn_2_S_4_ nanosheets (DZIS) dual‐functional photocatalysts (DZIS/WCQDs), which exhibit excellent hydrogen production rate and BAD selectivity in photocatalytic water splitting coupled with BA oxidation. First, WC with metallic like properties can form Schottky junctions with ZnIn_2_S_4_, and its combined effect with defects significantly improves the photogenerated carrier separation efficiency of DZIS/WCQDs composites. Second, WC with local surface plasmon resonance (LSPR) effect can effectively promote carrier mobility and reduce the energy barrier of the catalytic reaction through the photothermal effect, and thus greatly increasing the hydrogen production rate.^[^ ^14^ ^]^ Moreover, the photothermal effect can slowly induce the decomposition of H_2_O_2_ to produce ·OH,. It is worth noting that the low concentration ·OH and photogenerated holes continuously generated in situ can directly and rapidly attack the αC─H bond of BA to improve BA conversion rate and BAD selectivity.^[^ ^15^ ^]^ Third, the unsaturated eg orbitals can effectively capture photogenerated electrons from ZnIn_2_S_4_, thereby further promoting effective separation of electrons and holes, and prolonging carrier lifetime. In addition, S vacancies and WC can shift the d‐band center of the catalyst upward, resulting in strengthening the interaction between the adsorbed BA and the catalyst, and reducing the energy barrier for surface BA adsorption/activation/oxidation.^[^ ^16^ ^]^ As a result, DZIS/WCQDs composites exhibit a surprising conversion rate and selectivity of 85.34% and 96.53% for BAD, and outstanding H_2_ and BAD evolution rates of 12.58 and 10.53 mmol g^−1^ h^−1^ without sacrificial agent and co‐catalyst, which is one of the best reported photocatalysts. Finally, the high catalytic activity and selectivity of DZIS/WCQDs composites are demonstrated through a series of in situ characterizations and density functional theory (DFT) calculations. This work provides a new strategy for achieving efficient hydrogen production while providing a green synthesis pathway for the synthesis of industrial chemicals.

## 2 Results and Discussion

### 2.1 Morphological and Structural Characterization

**Figure 1:**
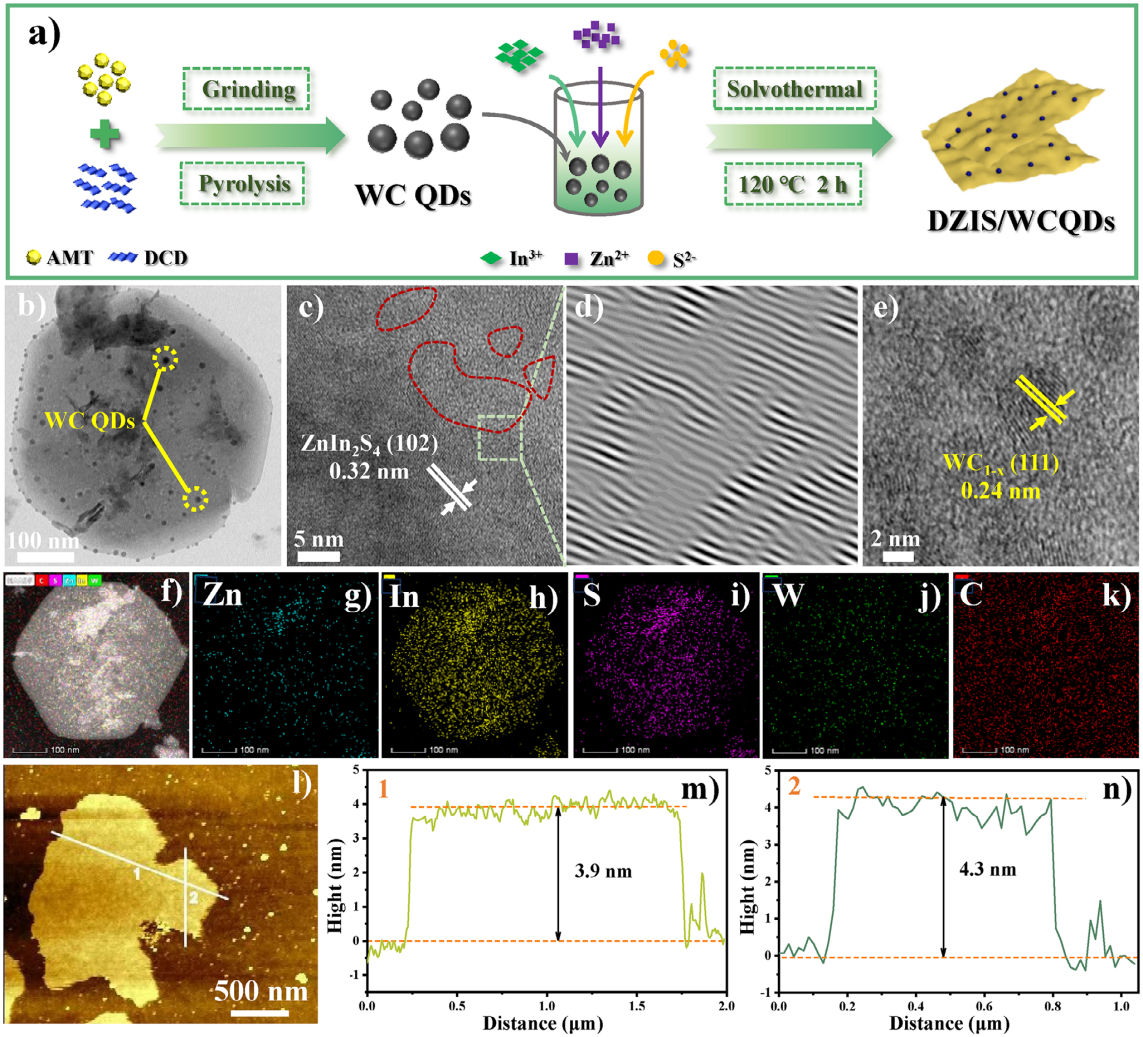
a) Schematic illustration of the synthesis process of DZIS/WCQDs. b–e) TEM and HRTEM images of DZIS/WCQDs. f–k) Elemental mapping images of Zn, In, S, W and C elements from DZIS/WCQDs. l–n) AFM image and the corresponding height profiles of DZIS/WCQDs.

The synthesis process of DZIS/WCQDs composites is illustrated in **Figure** **1**a, where WC QDs is used to modify DZIS. The morphology and thickness of the prepared samples were characterized by transmission electron microscopy (TEM) and atomic force microscopy (AFM). As shown in Figures S1 and S2 (Supporting Information), pure DZIS exhibits a nanosheet morphology with the average thickness of about 4.1 nm, and the surface shows obvious mesopores and disordered structures, indicating that there are amount defects on the surface of pure DZIS nanosheets, which not only conducive to the separation of photogenerated carriers, but also provides more active sites for catalytic reactions. Figure S3 (Supporting Information) shows that WC QDs is surrounded by amorphous carbon layers, and the size of WC QDs is approximately 2–5 nm. According to the thermogravimetric curve (Figure S4, Supporting Information), the content of carbon in WC QDs is 11.38%, and this carbon layer can effectively prevent the oxidation of WC QDs.^[^ ^17^ ^]^ The TEM image of DZIS/WCQDs composites show that WC QDs with a diameter of about 5–10 nm (consisting of several pure WC QDs with a diameter of 2–5 nm) are uniformly dispersed on the surface of DZIS nanosheets (Figure 1b). The high‐resolution TEM (HRTEM) images of DZIS/WCQDs (Figure 1c,e) show two lattice spacings of 0.32 and 0.24 nm, which correspond to the (102) crystallographic plane of ZnIn_2_S_4_ and the (111) crystallographic plane of WC_1‐_ *_x_*, respectively.^[^ ^18^ ^]^ In addition, it also can be seen that DZIS/WCQDs retains the abundant disordered structures of DZIS nanosheets (red coils), and these disordered structures show obvious dislocations (Figure 1d), indicating that there are many active sites on the surface of DZIS nanosheets. The elemental mapping images also show the well‐defined spatial distribution of Zn, In, S, W and C elements (Figure 1f–k), which further prove the successful synthesis of DZIS/WCQDs composites. Finally, AFM image show that DZIS/WCQDs composites can still maintain its ultra‐thin nanosheet morphology, and the average thickness does not change significantly (Figure 1l–n).

**Figure 2:**
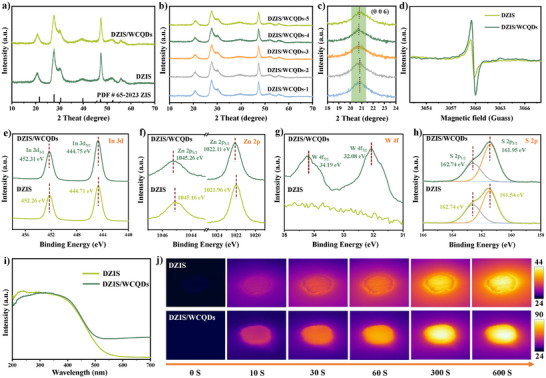
a) XRD patterns of DZIS, DZIS/WCQDs. b) DZIS/WCQs with different amount of WC QDs, and c) a partial enlarged view of (b). d–i) Low‐temperature EPR spectra, XPS spectra of Zn 2p, In 3d, S 2p, W 4f, and UV–vis absorption spectra of DZIS and DZIS/WCQDs. j) Infrared thermal imaging photographs of DZIS, DZIS/WCQDs and WC QDs under AM1.5G illumination.

The crystal structures of prepared samples were characterized via X‐ray diffraction (XRD). As shown in **Figure** **2**a, the diffraction peaks of pure DZIS are well indexed to the hexagonal phase ZnIn_2_S_4_ (PDF#65‐2023).^[^ ^19^ ^]^ The XRD pattern of WC QDs is shown in Figure S5 (Supporting Information), the characteristic peaks at 62.0° and 74.2° can be matched with the (220) and (311) planes of cubic WC_1‐_ *_x_* (PDF# 20‐1316),^[^ ^17c^ ^]^ respectively. The relative intensity of the peaks at 37.0° and 42.9° is different from the one in standard profile of WC_1‐_ *_x_*, which might be resulted from the preferential growth of (111) at the high carbon content. However, the characteristic diffraction peaks of WC_1‐_ *_x_* are not observed in DZIS/WCQDs composites, which may be due to the small size and load capacity of WC QDs.^[^ ^17^, ^20^ ^]^ It is worth noting that the (006) diffraction peak of DZIS shows a gradual blue shift as the load of WC QDs increases (Figure 2b,c), which indicates that there is obvious electron interaction between DZIS and WC QDs, and further proves the formation of Schottky junctions in DZIS/WCQDs. Low‐temperature electron paramagnetic resonance (EPR) was used to determine the presence of defects in DZIS and DZIS/WCQDs (Figure 2d). The results show that a strong signal at the Lorentzian line about *g* ≈ 2.003 can be detected, indicating that there are S defects in DZIS nanosheets.^[^ ^21^ ^]^ Furthermore, the signal of defect becomes stronger with the loading of WC QDs, suggesting that the defects of DZIS/WCQDs are increased.

X‐ray photoelectron spectra (XPS) was employed to identify the surface chemical structure and the interface interaction between DZIS and WC QDs, and the results were showed in Figure 2e–h and Figures S6 and S7 (Supporting Information). The prominent peaks at 1045.16 and 1021.96 eV in the Zn 2p XPS spectrum and 452.26 and 444.71 eV in the In 3d XPS spectrum of DZIS are ascribed to the Zn 2p_1/2_ and 2p_2/3_, and In 3d_3/2_ and 3d_5/2_, respectively. Besides, the peaks at 162.74 and 161.54 eV are assigned to the S 2p_1/2_ and 2p_3/2_.^[^ ^22^ ^]^ It is worth noting that the binding energy positions of Zn 2p and In 3d in DZIS/WCQDs composites exhibit varying degrees of redshift compared with DZIS nanosheets. In addition, the S 2p_3/2_ of DZIS preferentially bonds with W 4f, resulting in an increase in the binding energy of the spin splitting peak of S 2p_3/2_. The above results prove that there is a significant electron transfer between DZIS and WC QDs in DZIS/WCQDs. Moreover, the W 4f and C‐W peaks are also observed in the W 4f_7/2_ and C1s XPS spectra of DZIS/WCQDs composites, which proves that WC QDs is successfully loaded on the surface of DZIS nanosheets.^[^ ^23^ ^]^ N_2_ adsorption‐desorption isotherms and pore size distribution curves can be used to characterize the structural properties of materials. As shown in Figures S8 and S9 and Table S1 (Supporting Information), the N_2_ adsorption–desorption isotherms of DZIS and DZIS/WCQDs show type IV isotherm, and the relative pressure (*p*/*p*_0_) of hysteresis loop is 0.4 to 1.0, which proves that DZIS and DZIS/WCQDs have mesoporous structure.^[^ ^24^ ^]^ The specific Brunauer–Emmett–Teller surface area, pore size, and pore volume of DZIS/WCQDs are 68.28 m^2^ g^−1^, 11.01 nm and 0.16 cm^3^ g^−1^, respectively, which is much higher than that of DZIS. The large specific surface area of DZIS/WCQDs with 2D hierarchical structure may expose a large number of adsorption and actives sites for catalytic reactions.

The optical properties of the photocatalysts were investigated by UV–vis absorption spectra.^[^ ^25^ ^]^ The results are shown in Figure 2i and Figures S10–S12 (Supporting Information), pure DZIS nanosheets can only absorb visible light with wavelengths shorter than 600 nm, while DZIS/WCQDs composites exhibit significant light absorption in visible and near‐infrared regions, and absorption intensity gradually increases with the increase of WC QDs loading. The light absorption in the visible and near infrared is due to the fact that tungsten carbide is similar to precious metals with high free electron density, which can collectively oscillate under light conditions and produce LSPR effect.^[^ ^26^ ^]^ The light absorption in the near infrared region is more easily converted into heat energy to raise the surface temperature of the catalyst, which is beneficial for enhancing the photocatalytic activity of DZIS/WCQDs composites.^[^ ^27^ ^]^ Next, the photothermal conversion ability of samples was tested by an infrared thermal thermography instrument.^[^ ^28^ ^]^ As shown in Figure 2j and Figure S13 (Supporting Information), the temperature of DZIS nanosheets gradually increases under the light irradiations, and the surface temperature is about 44 °C after 10 min of illumination, which is attributed to non‐radiative recombination. Compared with DZIS, the temperature rise of DZIS/WCQDs composites is more significant under the same conditions, and the temperature gradually increases with the increase of WC QDs amount, reaching a maximum of 90 °C. The results further indicate that WC QDs have good photothermal conversion ability, and can be used as “heat island” to increase the surface temperature of DZIS/WCQDs.

### 2.2 Photocatalytic Performance Measurement

**Figure 3:**
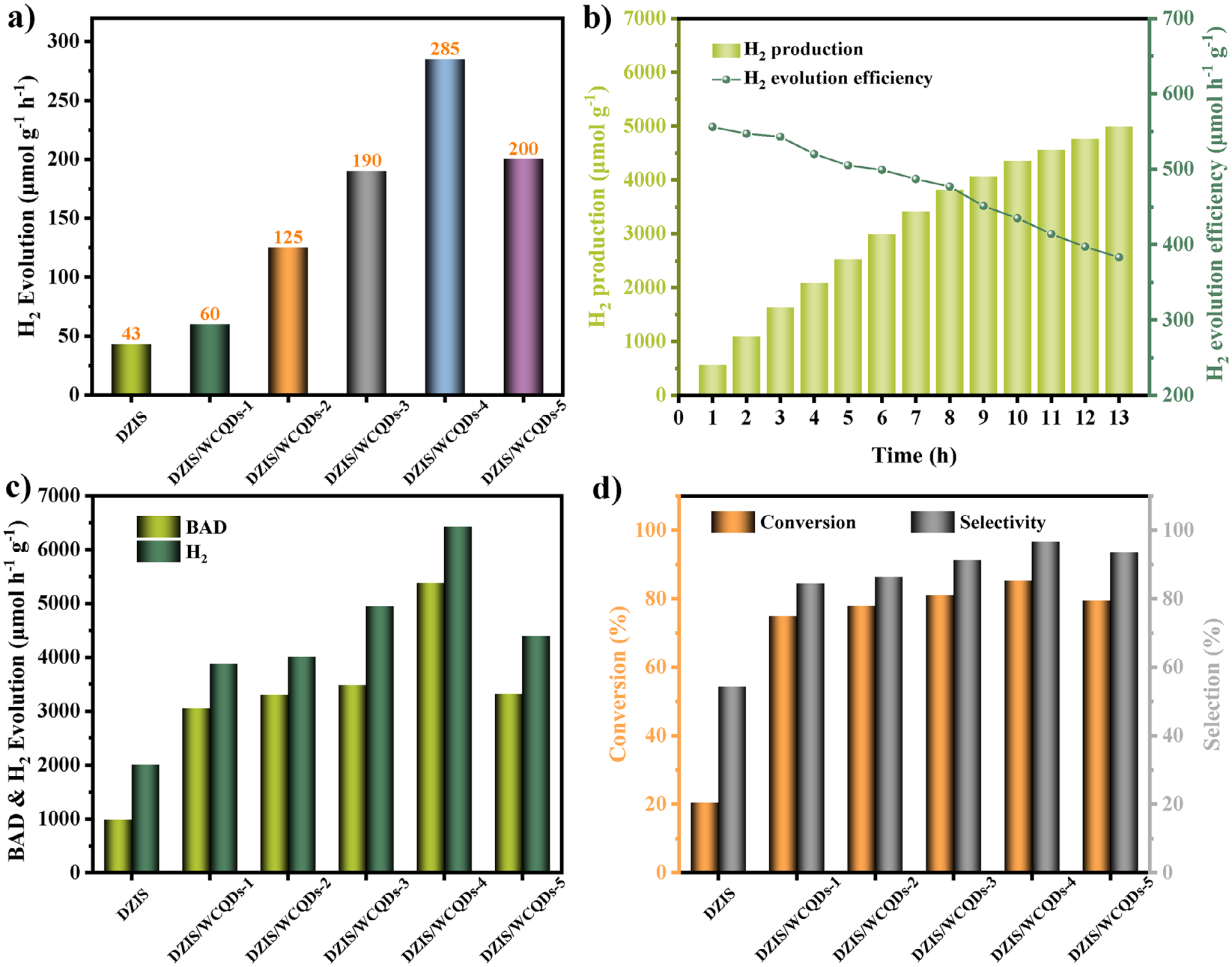
a) Photocatalytic pure water splitting for H_2_ evolution rates of DZIS and DZIS/WCQDs‐*n*. b) Time course of H_2_ evolution (left axis) and evolution efficiency (right axis) of DZIS/WCQDs. c) BAD and H_2_ evolution rates, d) conversion and selectivity of BAD after 10 h of solar irradiation of DZIS/WCQDs.

The photocatalytic activities of all samples were evaluated under simulated sunlight irradiation (AM 1.5G). As shown in **Figures** **3**a,b and S14 (Supporting Information), highly efficient H_2_ evolution (285 µmol g^−1^ h^−1^) and a small amount of H_2_O_2_ are detected in the overall pure water splitting of DZIS/WCQDs (corresponding to DZIS/WCQDs‐4) with the best WC QDs amount, and it is much higher than that of DZIS nanosheets (43 µmol g^−1^ h^−1^). However, the H_2_ evolution performance of DZIS/WCQDs composites slowly decreased with the extension of irradiation time. The results of the in‐situ electron spin resonance (ESR) show that DZIS/WCQDs composites can produce ·O_2_^−^ in methanol solution with the presence of O_2_ under illumination, which proves that the photocatalytic reaction includes water oxidation process with four‐electron and oxygen reduction process with two‐electron. (Figure S15, Supporting Information).^[^ ^5b^ ^]^ It is worth noting that the process of oxygen reduction requires the consumption of photogenerated electrons, which is a competitive reaction with water reduction to H_2_, and thus the DZIS/WCQDs composites shows a decrease in H_2_ evolution performance over time during the overall water splitting experiment. In addition, DZIS/WCQDs composites exhibit a stronger Lorentz signal than DZIS nanosheets (Figure S16, Supporting Information), proving that DZIS/WCQDs composites can produced more photogenerated electrons.

The four‐electron water oxidation process is the rate determining step of overall water splitting, and its slow reaction rate greatly limits the H_2_ evolution rate.^[^ ^6^ ^]^ Compared with the four‐electron water oxidation, the dehydrogenation of BA (two‐electron process) is more favorable kinetically.^[^ ^29^ ^]^ Therefore, using BA as water oxidation promoter can effectively improve the H_2_ evolution rate. In addition, the selective oxidation of BA can provide a green path for industrial production of BAD, and solve the problems of resource waste and environmental pollution in traditional sacrificial agent water splitting system.^[^ ^6^ ^]^ The performance and selectivity of photocatalytic water splitting coupled with BA oxidation without the addition of other sacrificial agents and cocatalysts are tested by high performance gas chromatography and liquid chromatography, and the results are shown in Figure 3c,d and Figures S17–S19 (Supporting Information). The photocatalytic water splitting coupled BA oxidation activities of DZIS/WCQDs composites are related to the loading amount of WC QDs and the concentration of BA. The H_2_ evolution rate of DZIS/WCQDs‐4 can reach maximum values of 12.58 mmol g^−1^ h^−1^ with the optimal BA concentration, which is much higher than that of pure DZIS (0.29 mmol g^−1^ h^−1^ of H_2_ evolution rate) under the same conditions. The average H_2_ evolution rate of DZIS/WCQDs is about 43.4 times than that of DZIS nanosheets. When the amount of BA exceeds the optimal value, the decrease in performance may be due to excessive BA hindering light absorption, occupying hydrogen producing active sites, and reacting with photogenerated electrons or intermediate products.^[^ ^30^ ^]^ In addition, DZIS/WCQDs composites also exhibit a surprising BAD evolution rates of 12.58 and 10.53 mmol g^−1^ h^−1^, and the conversion rate and selectivity of 85.34% and 96.53% for BAD after 5 h. It is worth noting that DZIS/WCQDs is the optimal catalyst material combining the H_2_ production rate, the conversion rate and selectivity of BA at present (Table S3, Supporting Information).

The photocatalytic H_2_ evolution performances have been further investigated under different oxidizing half‐reaction conditions for comparison. DZIS/WCQDs composites show the H_2_ evolution rates of 1.95 and 3.34 mmol g^−1^ h^−1^ with Na_2_S/Na_2_SO_3_ and lactic acid as sacrificial agents (Figure S20, Supporting Information). Excitingly, the H_2_ evolution rate of DZIS/WCQDs composite in BA solution is higher than that of other sacrificial agent systems, indicating that BA oxidative dehydrogenation is beneficial for efficient H_2_ production, and also proving its advantage in eliminating the use of sacrificial agents in photocatalysis. Next, the apparent quantum efficiency (AQE) of DZIS/WCQDs composites in pure water was measured with different monochromatic filters to evaluate the catalytic performance under different incident light. According to Figure S21 (Supporting Information), the AQE of DZIS/WCQDs follows the light absorption trend, which proves that the photocatalytic reaction is driven by photon excitation. The stability of DZIS/WCQDs composites were evaluated by repetitive photocatalytic experiments. As shown in Figure S22 (Supporting Information), the H_2_ evolution rate of the DZIS/WCQDs composites only decreases slightly after 5 cycles of experiments, indicating its good photocatalytic stability. In addition, the catalysts used in the cycle test were characterized by TEM, XRD and XPS (Figures S23–S25, Supporting Information). The results show that there is no significant difference in the morphology of DZIS/WCQDs before and after the cyclic experiment, and the XRD pattern and XPS spectra also show almost identical peaks, implying that DZIS/WCQDs composites have sufficient structural stability.

### 2.3 Photocatalytic Mechanism Investigation

**Figure 4:**
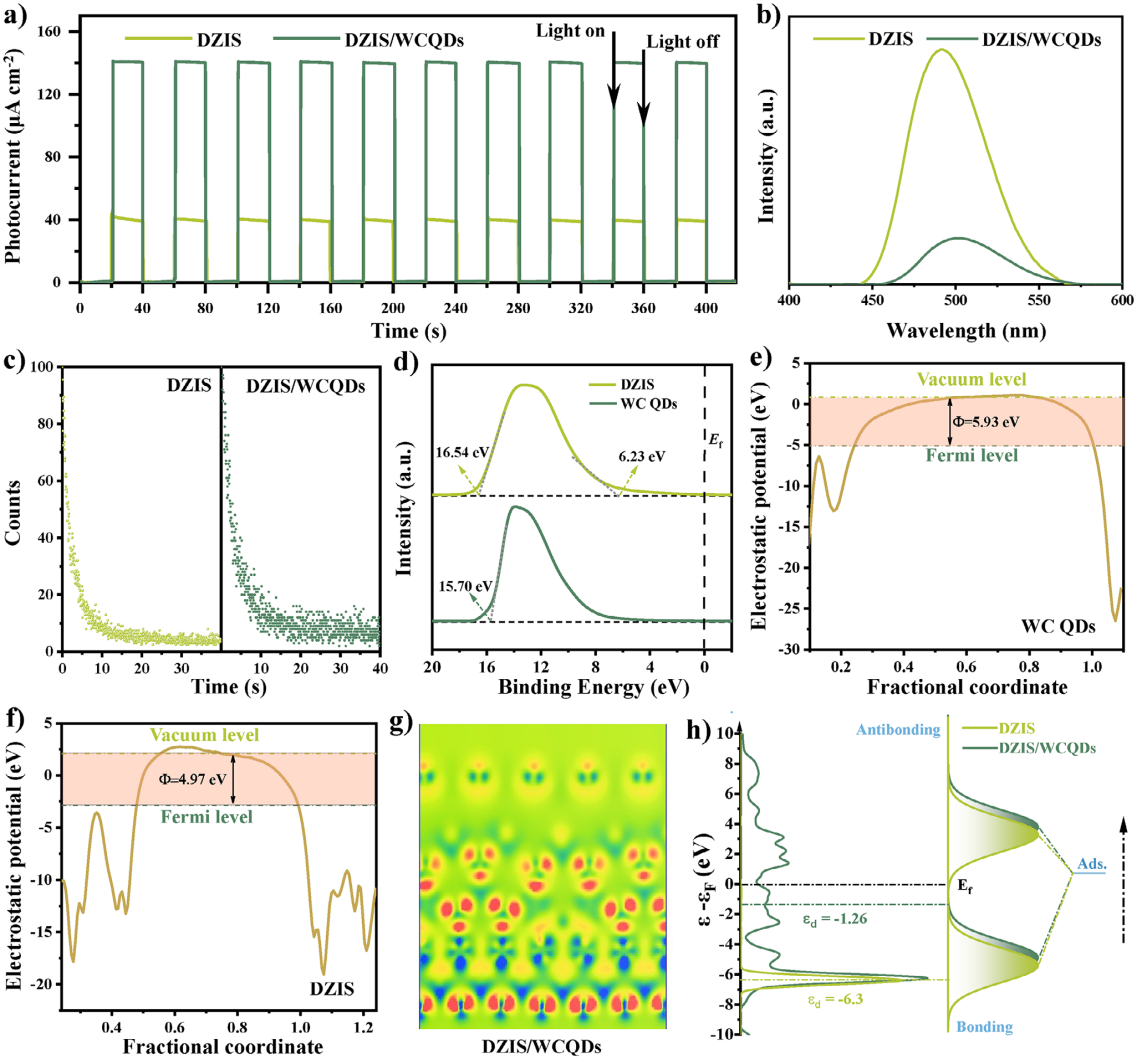
a–c) Photocurrent responses, photoluminescence spectra, time‐resolved fluorescence spectra of DZIS and DZIS/WCQDs. d) UPS spectra of DZIS and WC QDs. e) The calculated work function and corresponding structural model of the (111) planes of WC QDs and f) the calculated work function and corresponding structural model of the (102) planes of DZIS. g) Differential charge density of DZIS/WCQDs. h) Diagram of d‐band center shift of DZIS and DZIS/WCQDs.

In order to investigate the separation and recombination efficiency of photogenerated electrons and holes, photoelectric experiments were carried out. As shown in **Figure** **4**a, when the lamp is turned on, catalysts will excite photogenerated electrons immediately, and the photocurrent density of DZIS/WCQDs composites (139 µA cm^−2^) is 3.5 times than that of pure DZIS nanosheets (40 µA cm^−2^). Moreover, photocurrent densities of DZIS/WCQDs and DZIS have not decreased after 10 cycles, proving both catalysts have good photoelectric stability, and DZIS/WCQDs has more photogenerated electron‐hole pairs.^[^ ^31^ ^]^ From the electrochemical impedance spectroscopy (EIS) Nyquist diagrams of DZIS/WCQDs and DZIS (Figure S26, Supporting Information), the semicircle diameter of DZIS/WCQDs is smaller, indicating that DZIS/WCQDs has a lower interfacial charge transfer resistance.^[^ ^32^ ^]^

The photoluminescence (PL) is usually used to reflect the recombination situation of generated carriers. As shown in Figure 4b, the PL spectra of DZIS/WCQDs and DZIS both have strong emission peaks in the range of 440 to 570 nm, which are caused by the bandgap transition of DZIS.^[^ ^31a^ ^]^ Furthermore, the PL intensity of DZIS/WCQDs significantly decreases, suggesting that the charge recombination in DZIS/WCQDs is efficiently suppressed by Schottky junction. In addition, the migration dynamics of photoinduced carriers are investigated through ultrafast transient absorption (TA) spectra. The results are shown in Figure 4c and Table S2 (Supporting Information), according to the calculation, the average PL lifetime of DZIS/WCQDs composites (8.56 ns) is much longer than that of DZIS nanosheets (5.33 ns), suggesting that DZIS/WCQDs has a higher participation probability and priority in the photocatalytic reaction.

Next, the ultraviolet photoelectron spectroscopy (UPS) spectra were used to investigate the interface carrier transfer mechanism (Figure 4d). The results show that the work function of DZIS and WC QDs can be deduced of 4.68 and 5.52 eV versus vacuum level, respectively, and the valance band maxim of DZIS is 6.23 eV. Therefore, WC QDs can be used as a cocatalyst to form Schottky junctions with DZIS to improve the photocatalytic activity. In order to further investigate the interface charge transfer path of DZIS/WCQDs composite, the electronic properties are calculated by systematic density functional theory (DFT). The constructed models of DZIS, WC_1‐_ *_x_* and DZIS/WCQDs were established (Figure S27, Supporting Information). As shown in Figure 4e,f, the work functions of the (102) plane of DZIS and (111) plane of WC are calculated to be 4.97 and 5.93 eV versus vacuum level, which is slightly higher than the results of UPS test. It is worth noting that the fully occupied d‐orbitals are the essential features of Zn and In atoms (3d^10^), hence the 2p orbitals of C or O atoms in BA usually form weak bons with the d orbitals of Zn and In atoms. This weak interaction makes the efficiency of photocatalytic BA oxidation reaction still unsatisfactory for ZnIn_2_S_4_. The outermost layer orbital of the W atom in WC is the 5d^4^ structure, which can undergo effective crystal field splitting. Therefore, when WC is recombined with ZnIn_2_S_4_, the unsaturated eg orbitals of the W atoms can effectively capture photogenerated electrons from ZnIn_2_S_4_, and thus promoting effective separation of photogenerated electrons and holes, and prolonging carrier lifetime. The differential charge density of DZIS/WCQDs also confirms this conclusion (Figure 4g), and the electron density significant increases around WC, which is beneficial for the adsorption of H^+^.^[^ ^33^ ^]^ In addition, DFT calculations show that S vacancies and WC can shift the d‐band center of the DZIS/WCQDs upward (Figure 4h and Figure S28, Supporting Information), resulting in strengthening the interaction between the adsorbed BA and the catalyst, and reducing the energy barrier for surface BA adsorption/activation/oxidation. The above conclusions further prove that DZIS/WCQDs composites may exhibit excellent photocatalytic activity of water splitting coupled with BA oxidation from a theoretical perspective.

**Figure 5:**
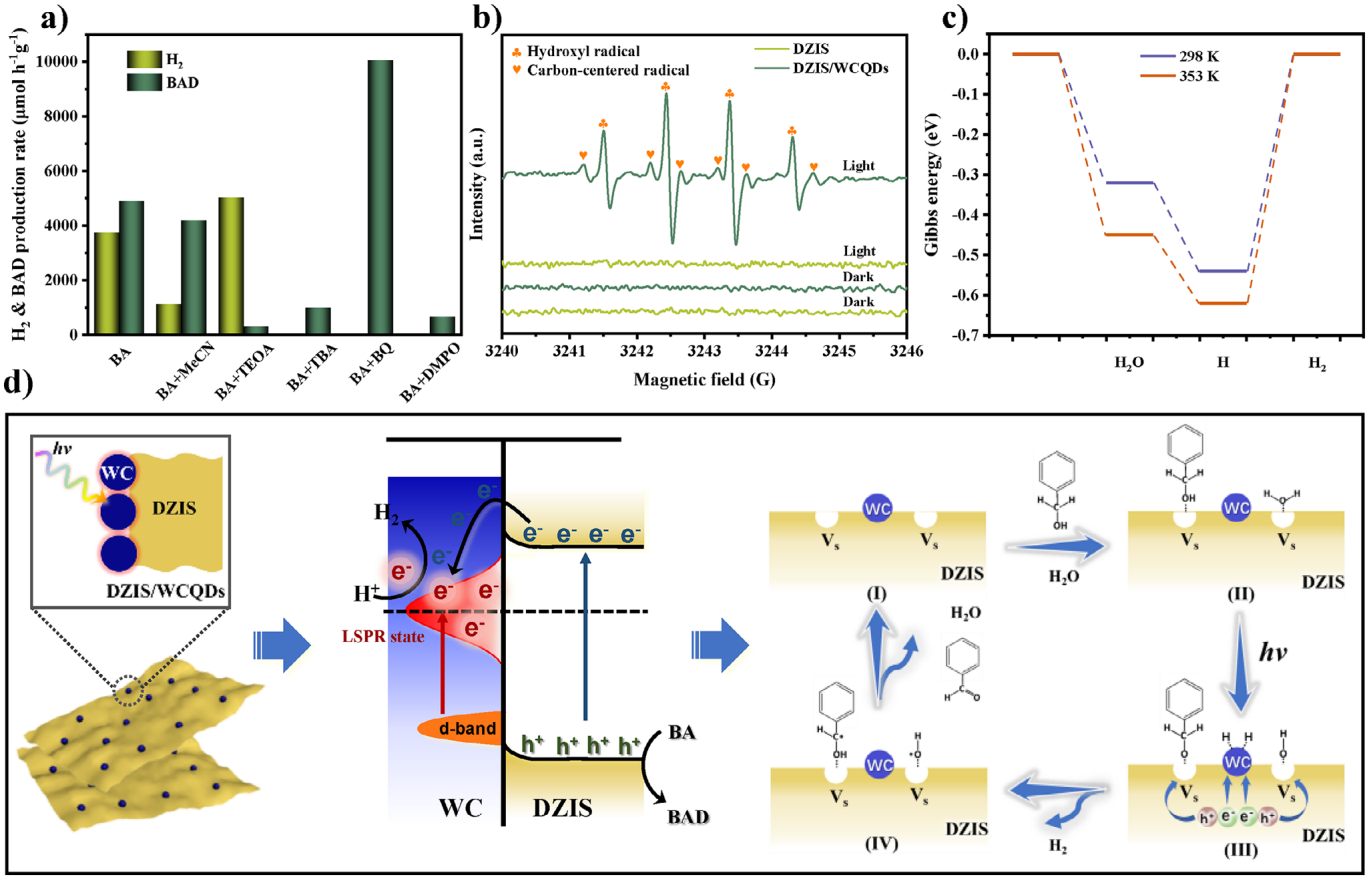
a) Effects of different radical quenchers on photocatalytic performance toward the H_2_ evolution and photocatalytic oxidation of BA over DZIS/WCQDs. b) In‐situ ESR detection of DZIS/WCQDs with or without light irradiation. c) Gibbs energy of photocatalytic reaction with different temperatures over DZIS. d) Schematics of DZIS/WCQDs for solar‐driven photothermal synergistic catalytic water splitting coupled with selective BA oxidation.

It is well known that photocatalytic oxidation is a kind of free radical reaction, and thus a series of free radical capture control experiments are carried out to explore the reaction mechanism of oxidative dehydrogenation of BA. As shown in **Figure** **5**a, first, anhydrous acetonitrile is used instead of water as the reaction solvent, the results show that both H_2_ and BAD are reduced, proving that the source of protons is water and the oxidative dehydrogenation of BA, and water has a promoting effect on BA oxidation. Next, the tertiary butanol (TBA) is selected as scavenging agent to capture ·OH, and the result shows that the yield of BAD decreases significantly, which further proves that ·OH is one of the main active species in BA oxidation.^[^ ^34^ ^]^ Triethanolamine (TEOA) is commonly used as a trapping agent for photogenerated holes. The BAD production significantly decreases after adding TEOA, whereas H_2_ production increases slightly, which proves that photogenerated holes and ·OH jointly drive the BA transformation.^[^ ^35^ ^]^ The introduction of 5,5‐dimethyl‐1‐pyrroline‐Noxide (DMPO), a carbon‐centered radical scavenger, can study the possible intermediate processes. Under this condition, the amount of BAD still decreases significantly, indicating that carbon‐centered radical is the main intermediate of BA conversion. 1,4‐benzoquinone (BQ) is usually used as a scavenger of ·O^2−^,^[^ ^36^ ^]^ but when its concentration is too high, it can consume a large amount of photogenerated electrons in a short period of, thereby inhibiting the production of H_2_. Moreover, the amount of BAD do not decrease, and thus the role of ·O^2−^ in the oxidation process of BA can be ignored.^[^ ^37^ ^]^ Subsequently, in situ ESR spectra were collected at room temperature in an aqueous solution of BA with DMPO as spin trapping reagent under Ar atmosphere. As shown in Figure 5b, DZIS has no characteristic signal peak under both dark and light conditions, while DZIS/WCQDs can observe significant ESR signals of ·OH and carbon‐centered radical under light condition.^[^ ^38^ ^]^ The carbon‐centered radical is considered to be the key intermediate for BA dehydrogenation, and thus further proving that loading WC QDs can promote photocatalytic BA dehydrogenation to BAD.

DZIS/WCQDs photocatalytic overall water splitting can produce H_2_ and H_2_O_2_, and H_2_O_2_ will decompose into ·OH under light and high temperature, which is also the reason for the low H_2_O_2_ content in the overall water splitting process, and the presence of ·OH in the free radical capture experiment and ESR test results. Coumarin is selected as a fluorescent probe to test the ·OH concentration changes of DZIS/WCQDs, due to coumarin can react with ·OH to generate 7‐hydroxycoumarin with strong properties. Figure S29 (Supporting Information) shows that DZIS hardly produces ·OH under illumination, while the ·OH concentration of DZIS/WCQDs composites increases with the increase of illumination time, which is consistent with the ESR results. However, the concentration remains at a low level after 4 h of illumination (Figure S30, Supporting Information). It is worth noting that the high concentration of ·OH can lead to further oxidation of BAD to benzoic acid, and thus low concentration of ·OH is crucial for enhancing the selectivity of BAD. This result proves that the photothermal effect of WC QDs can slowly induce the decomposition of H_2_O_2_ to produce ·OH, and the low concentration ·OH and photogenerated holes continuously generated in situ can directly and rapidly attack the αC─H bond of BA, resulting in DZIS/WCQDs exhibiting superior BAD conversion rate and selectivity compared to DZIS.

The conduction band of DZIS and WC QDs as −0.67 and ‐0.57 eV versus NHE (pH = 7) can be obtained according to Mott‐Schottky (M‐S) plots (Figure S31, Supporting Information), and the Fermi level of DZIS and WC QDs as −0.01 and 0.83 eV versus NHE (pH = 7) can be deduced according the work functions by UPS and DFT. Based on the above results, we can obtain the band structure diagram of DZIS and WC QDs as shown in Figure S32 (Supporting Information), and prove the formation of Schottky junction in DZIS/WCQDs. In addition, the results show that the valence band of DZIS can achieve the oxidation of BA to BAD, but cannot meet the oxidation transition point for further oxidation of BAD to benzoic acid. Therefore, DZIS/WCQDs composites have excellent selectivity for BAD. Finally, in order to demonstrate the promotion of photothermal effect on H_2_ evolution activity, we calculated the Gibbs free energy (Δ*G*) of H_2_ evolution reaction with different temperature using DFT. As shown in Figure 5c, the Δ*G* for the first and second steps are −0.323 and ∓0.544 eV at 298 K in the H_2_ evolution reaction process; these steps correspond to H_2_O adsorption and H adsorption, respectively. When the temperature is enhanced to 353 K, the system exhibits lower Δ*G* values of −0.452 and −0.622 eV for the H_2_O and H adsorption steps, respectively, revealing more favorable thermodynamics for H_2_ evolution reaction, which is consistent with our experimental results.

Herein, the excellent photocatalytic performance can be attributed to the nanoscale architectural design of the DZIS/WCQDs Schottky junction, which can be used as an integrated system to achieve efficient hydrogen production and high value‐added chemical conversion in broad solar spectrum. The mechanism of photothermal synergistic catalytic water splitting coupled with selective BA oxidation of DZIS/WCQDs is proposed in Figure 5d. i) The defect structure on the surface of DZIS nanosheets can not only significantly improves the separation efficiency of photogenerated carrier, but also facilitate the adsorption of BA. ii) In DZIS/WCQDs composites, WC QDs can form a Schottky junction with DZIS, allowing photogenerated electrons to immediately transfer to the surface of WC QDs. Then, these photogenerated electrons participate in the photocatalytic proton reduction process together with the hot electrons generated by the LSPR effect of WC QDs. iii) It is noteworthy that low concentration ·OH and photogenerated holes with moderate oxidation ability can directly and rapidly attack the αC‐H bond of BA to improve BA conversion rate and BAD selectivity. iv) The photothermal effect of WC QDs can accelerate the migration of photogenerated carriers and reduce the potential barrier of catalytic reduction reaction through increasing the surface temperature of the material, resulting in outstanding H_2_ evolution rates and excellent conversion rate and selectivity for BA oxidation.

## 3 Conclusion

In summary, we have reported the synthesis of a novel WC QDs decorated defective ZnIn_2_S_4_ nanosheets (DZIS/WCQDs) photocatalysts with outstanding activity and selectivity of water splitting coupled with selective BA oxidation. The enhanced photocatalytic performance and selectivity can be attributed to the following aspects. First, the surface defects and Schottky junction of DZIS/WCQDs can significantly improve the separation efficiency of photogenerated carriers. Secondly, the band structure of DZIS/WCQDs determines low concentration ·OH and photogenerated holes with moderate oxidation ability, which can ensure the high selectivity of BAD. Finally, WC QDs with the LSPR effect can generate a large number of high‐energy hot electrons to directly participate in H_2_ evolution reaction, and the excellent photothermal performance can reduce the activation energy barrier for catalytic reduction and accelerate the transmission of photogenerated carriers, thereby enhancing photocatalytic activity. This work provides a novel experimental strategy for achieving high photocatalytic H_2_ evolution rate while providing a green synthetic pathway for industrial chemicals.

## 4 Experimental Section

All pertinent details can be found in the Supporting Information.

## Conflict of Interest

The authors declare no conflict of interest.

## Supporting information



Supporting Information

## Data Availability

The data that support the findings of this study are available from the corresponding author upon reasonable request.
