# Supplementary material for: Utilizing Photothermal Effect Enhances Photocatalytic Water Splitting Coupled with Selective Benzyl Alcohol Oxidation over Schottky Junctions
Source: Adv Sci (Weinh). 2025 Apr 11;12(26):2501931. doi: 10.1002/advs.202501931 (PMC12245034; doi:10.1002/advs.202501931)
Supplement: Supplementary file 1 — Supporting Information [file ADVS-12-2501931-s001.docx]

**Supporting Information**

**Utilizing Photothermal Effect Enhances Photocatalytic Water Splitting Coupled with Selective Benzyl Alcohol Oxidation Over Schottky Junctions**

Bojing Sun^a,b,d^, Mengjia Ye^a^, Yachao Xu^c^*, Ying Jiang^a^, Dongfang Hou^a,d^, Xiu-qing Qiao^a,d^, Yunchen Du^b^*, and Dong-Sheng Li^a,d^*

*^a^ College of Materials and Chemical Engineering, Key Laboratory of Inorganic Nonmetallic Crystalline and Energy Conversion Materials, China Three Gorges University, Yichang, Hubei 443002, P.R. China*

*^b^ MIIT Key Laboratory of Critical Materials Technology for New Energy Conversion and Storage, School of Chemistry and Chemical Engineering, Harbin Institute of Technology, Harbin 150001, China.*

*^c^ School of Materials Science and Engineering, Peking University, Beijing 100871, China*

*^d^ Hubei Three Gorges Laboratory, Yichang, Hubei 443007, P. R. China*

* *Corresponding authors*.

E-mail address: xyc@pku.edu.cn (Yachao Xu); yunchendu@hit.edu.cn (Yunchen Du); lidongsheng1@126.com (Dong-sheng Li).

**Experimental section
Materials**All chemicals were used without further purification. Ammonium metatungstate (AMT, H_28_N_6_O_41_W_12_), indium chloride tetrahydrate (InCl_3_·4H_2_O), zinc chloride (ZnCl_2_) and thioacetamide (TAA, C_2_H_5_NS) were purchased from Shanghai Macklin Bio-Chem Technology Co., Ltd. dicyandiamine (DCD, C_2_H_4_N_4_) and benzyl alcohol were purchased from Sinopharm Chemical Reagent Co., Ltd.

**Synthesis of WC QDs**

Firstly, 0.3 g of dicyandiamine and 0.9 g of ammonium metatungstate were thoroughly ground in an agate motar for 20 mins. The resultant solid mixture was then pyrolyzed at 400 ℃ for 0.5 h, then kept at 800 ℃ for 5 h under N_2_ atmosphere, and the flow rate of N_2_ was controlled at 2.5 mL/min. The heating rates from room temperature to 400 ºC and from 400 ºC to 800 ºC were 2 ºC/min and 5 ºC/min, respectivley. The products were denoted as WC QDs.

**Synthesis of DZIS**

ZnIn_2_S_4_ nanosheets were prepared using a solvothermal method under magnetic stirring. In detail, 140 mg of ZnCl_2_, 590 mg of InCl_3_·4H_2_O and 310 mg of TAA were added to 20 mL of methyl alcohol and stirred for 1 h. Subsequently, the solution was transferred to a 40 mL autoclave, and heated to 120 ℃ for 2 h under magnetic stirring. After the system was cooled, the dispersion was washed with water and ethanol, and then vacuum dried at 60 ℃ for 4 h. The resultant sample represented the DZIS.

**Synthesis of DZIS/WCQDs**

Firstly, a certain amount of newly prepared WC QDs was added to 20 mL of methyl alcohol. After treating in an ultrasonic bath for 0.5 h, Then, 140 mg of ZnCl_2_, 590 mg of InCl_3_·4H_2_O and 310 mg of TAA were dispersed in sequence and stirred for 1 h. Subsequently, the solution was transferred to a 40 mL autoclave, and heated to 120 ℃ for 2 h under magnetic stirring. After the system was cooled, the dispersion was washed with water and ethanol, and then vacuum dried at 60 ℃ for 4 h. A yellow-green powder sample was obtained and named DZIS/WCQDs-*n*. WC QDs has a mass of 20 to 60 mg. DZIS/QDs-1, DZIS/QDs-2, DZIS/QDs-3, DZIS/QDs-4 and DZIS/QDs-5 correspond to 20 mg, 30 mg, 40 mg, 50 mg and 60 mg.

**Photocatalytic H_2_ evolution**

Photocatalytic hydrogen production was conducted in an online hydrogen production system (Perfect Light, Beijing, MicroSolar-300). Each of the photocatalysts (20 mg) was suspended in ultrapure water (80 mL). Prior to the reaction, the mixture was deaerated by evacuation to remove air dissolved in the water. The reaction was carried out under the irradiation of a xenon lamp (300 W) equipped with AM1.5 G transmission filter at constant filament current strength (15 A). Gas evolved was analyzed by an inline gas chromatograph (SP9700, TCD, molecular sieve 5 Å, N_2_ carrier, Beijing Keruida Limited). The determination of the apparent quantum efficiency (AQE) and solar-to-hydrogen (STH) efficiency for photocatalysis hydrogen generation were obtained under the xenon lamp equipped with single-wavelength filters (365, 420, 450, 520, 550, 650, 700 nm) at filament current intensity of 15 A. The calculation process refers to the classical calculation formula of AQE:

$AQE=\frac{2{N(H}_{2})}{N(Photos)}\times100\%$ (1)

where the N(H_2_) is the number of evolved H_2_ molecules, N(Photos) is the number of incident photos. Further, the number of hydrogen molecules can be expressed as:

${N(H}_{2})=n{(H}_{2})N_{A}$ (2)

And the number of incident photons can be expressed as

$N\left( Photos \right)=\frac{E\times\lambda}{h\times c}=\frac{F\times S\times T\times\lambda}{h\times c}$ (3)

where *n*(H_2_) refers to the hydrogen evolution (mol), *T* is the irradiation time (s), *N*_A_ is the *Avogadro constant* (6.022×10^23^ mol^-1^), *E* refers to the total energy of the incident photon (J), *F* refers to the average spectral irradiance (w/cm^2^), *S* is the irradiation area (69.4 cm^2^ in this paper), *λ* is the wavelength of monochromatic light (m), *h* is the *Planck constant* (6.626×10-^34^ J•s) and *c* is the light speed (3.0×10^8^ m/s^1^). All values are in SI units. By integrating formulas (1), (2) and (3), the AQE is obtained as follows:

$AQE=\frac{2\times n{(H}_{2})\times N_{A}\times h\times c}{F\times S\times T\times\lambda}100\%$ (4)

The STH efficiency can be calculated as follows:

$STH=\frac{output energy as H_{2} gas}{energy of incident solar light}=\frac{V_{H_{2}}\times\Delta G}{P_{sun}\times S}100\%$ (5)

where V_H2_ is the rate of hydrogen production (mmol/s), *P*_sun_ is energy flux of the sunlight (mW/cm^2^), *S* is the area of the reactor (69.4 cm^2^), and ΔG is the gain in Gibbs free energy (237 kJ/mol).

**Photocatalytic splitting of pure water**

The equipment used in photocatalytic decomposition of pure water to produce hydrogen is the same as that used in photocatalytic production of hydrogen (Perfect Light, Beijing, MicroSolar-300). The catalyst (DZIS/WCQDs, 20 mg) was dispersed in deionized water (80 ml) . xenon lamps were used to illuminate the solution (AM1.5, at room temperature). Gas samples are taken every hour by the inline gas chromatograph (SP9700, TCD, molecular sieve 5 Å, N_2_ carrier, Beijing Keruida Limited). The hydrogen evolution efficiency is calculated directly from each sampling result minus the sampling result of the previous hour. The oxidation product of pure water is not oxygen but H_2_O_2_ which was affirmed by a H_2_O_2_ concentration meter.

**Coupling of photocatalytic H_2_ evolution and phenylcarbinol oxidation**

The equipment used in photocatalytic coupling of photocatalytic H_2_ evolution and phenylcarbinol oxidation is the same as that used in photocatalytic production of hydrogen (Perfect Light, Beijing, MicroSolar-300). The preparation of the catalyst is the same as that of photocatalytic decomposition of pure water. After the ice had melted, Phenylcarbinol (104 μL) is added to the suspension, ultrasonic and stirring respectively for 10 minutes. Prior to the reaction, the mixture was deaerated by evacuation to remove air dissolved in the water. The reaction was carried out under the irradiation of a xenon lamp (300 W) equipped with AM1.5 transmission filter at constant filament current strength (15 Å). The temperature of the reaction system was controlled at 25 ℃ by injecting constant temperature water into the reactor sandwich. Gas evolved was analyzed by an inline gas chromatograph (SP9700, TCD, molecular sieve 5 Å, N_2_ carrier, Beijing Keruida Limited). The structure of the liquid product was characterized by comparing the retention time with the authentic compound of high performance liquid chromatography (HPLC).

**Photoelectrochemical measurements**Photoelectrochemical tests were performed on an electrochemical workstation (CHI660E) in 0.5 M Na_2_SO_4_ electrolyte solution. The standard three-electrode system consists of a counter electrode (Pt wire), a reference electrode (Hg/HgCl_2_ in saturated KCl) and a working electrode. The working electrode was prepared as follows: 4 mg of photocatalyst sample was dispersed in a mixture containing 700 μL of deionized water, 200 μL of anhydrous ethanol, and 100 μL of sodium fluoride. Then 60 μL of the mixture was drop-coated onto FTO glass (2 cm^2^) and dried naturally. The transient photocurrent response (I-t), Mott-Schottky (M-S) curves at different frequencies, and electrochemical impedance spectra (EIS) of the samples were tested in 0.5 M Na2SO4 electrolyte. The light source was a 300 W xenon lamp equipped (PLS-SXE300, Perfect light, China).

**Detection of reactive radicals**

The reactive radicals were detected by EPR measurements at room temperature using DMPO as the trapping agent. (10 mg catalyst, 4 ml H_2_O 1 ml BA, 30 μL DMPO) with nitrogen for 10 min and vacuumized it. Then filled it into capillary glass tube in a glove box (filled with argon), sealing both ends with vaseline. The signal of hydroxyl radical and superoxide radical can be detected by ESR.

**Calculation details**The DFT calculations have been carried out with the projector-augmented wave (PAW) method using the Perdew-Burke-Ernzerhof (PBE) exchange-correlation functional for the geometry optimizations and self-consistent total energy calculations. To describe the on-site Coulomb interaction among the localized zinc 3delectrons, we adopted the GGA+U approximation with an effective Hubbard U-parameter (Ueff = 8.5 eV). Calculations were performed with the Cambridge Sequential Total Energy Package (CASTEP); the plane wave cut off was set at 400 eV, and the total energy convergence at 10-6 eV for the self-consistent iterations. The Gaussian smearing method with σ = 0.05 eV was considered for Brillouin-zone integrations on a 3 × 3 × 1 k-mesh. The geometry optimizations were performed using a damped molecular dynamics scheme until the forces on the atoms were <0.01 eV/Å.

**Characterizations**

The phase and crystal structures of the samples were determined by X-ray power diffraction (P-XRD; Ultima IV, Japan) with Cu Kα radiation (λ=1.5418 Å) in the diffraction range from 5°-80°and the scanning speed was 8 °/min. The micro-structure and morphology of as-prepare photocatalyst were recorded on a JEOL-JSM-7500F instrument. Transmission electron microscopy analysis was characterized by Tecnai G2 F20 S-STWIN instrument at an operating voltage of 10 kv. The surface chemical valance and element composition of the samples were analyzed through X-ray photoelectron spectroscopy (XPS, Al-Kα) on an ESCALAB 250 equipment. The N_2_ adsorption-desorption isotherms of the samples were obtained by a Belsorp-max system. Which we can get the information of specific surface area and pore size distribution through Brunauer–Emmett–Teller (BET) and Barrett–Joyner–Halenda (BJH) analysis, respectively. The UV-vis diffuse reflectance spectroscopy (DRS) were carried out with a ultraviolet-visible spectrophotometer (PerkinElmer USA) with BaSO_4_ as a reflectance standard. The ptotoluminescence (PL) spectra and time-resolved spectrum were acquired by (PerkinElmer USA) instrument.


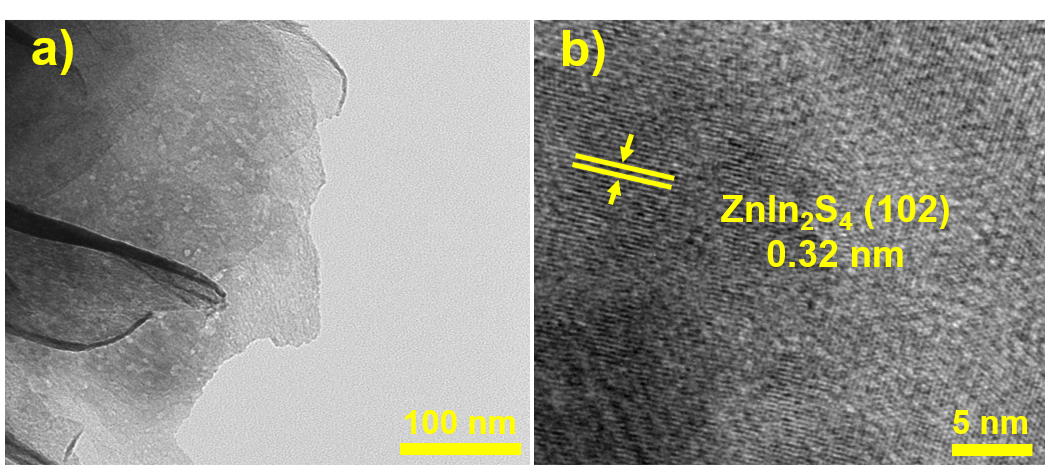


**Figure S1.** a) TEM and b) HRTEM images of DZIS


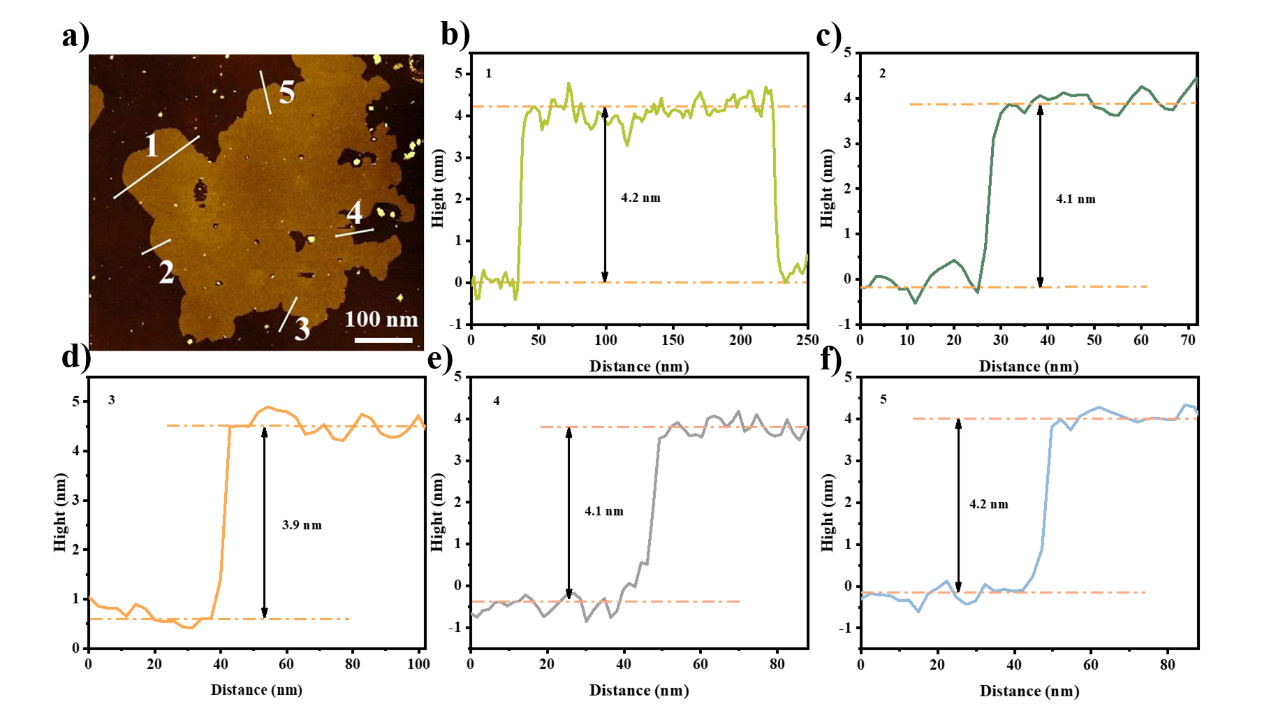


**Figure S2.** a) AFM image and b-f) energy dispersive spectroscopy mapping of DZIS.


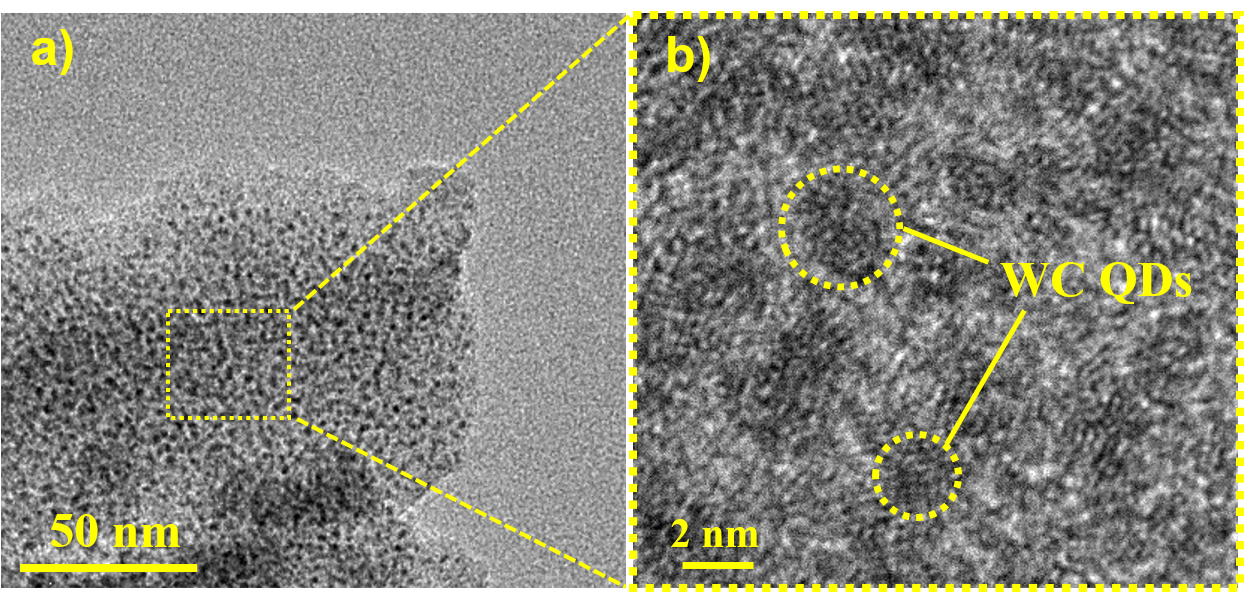


**Figure S3.** TEM images of WC QDs.


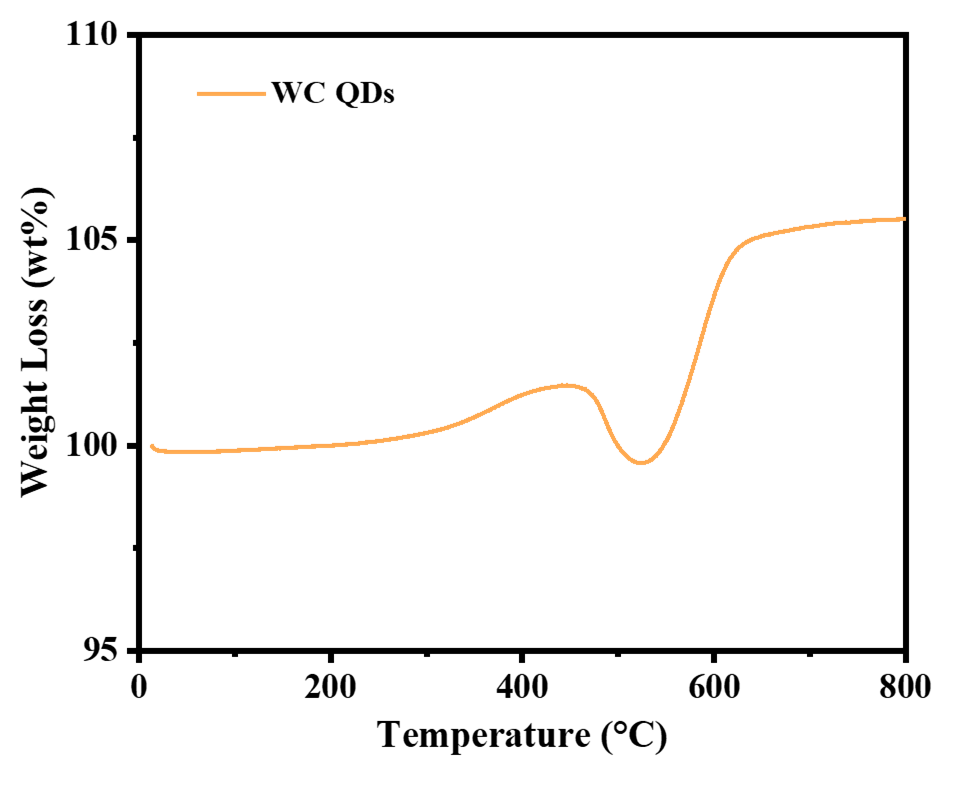


**Figure S4.** TGA curves of WC QDs nanosheets in air.

one can estimate the specific content of carbon nanosheets with equation:

$R wt\%=(1-C wt\%)\frac{M_{WO_{3}}}{M_{WC}}$ (6)

where *R* wt%, *C* wt%, *M*_WO3_, and *M*_WC_ are referred to remaining weight percentage, carbon nanosheets content, WO_3_ formula weight, and WC QDs formula weight.


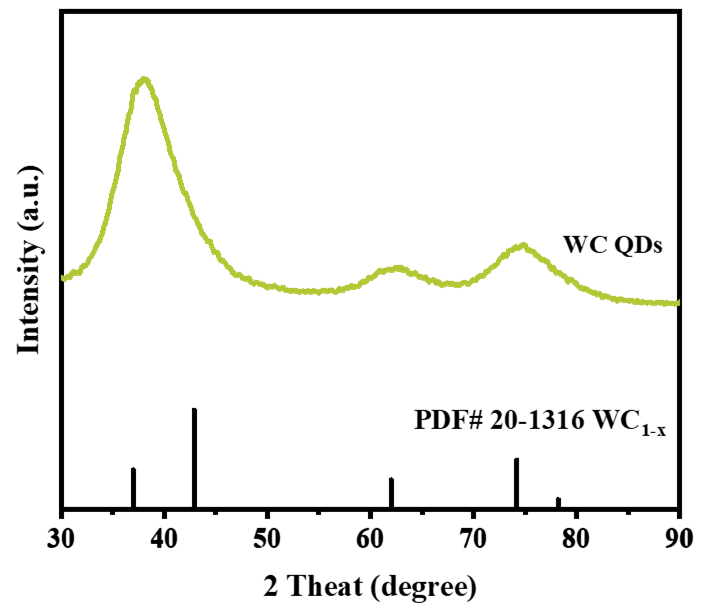


**Figure S5.** XRD patterns of WC QDs.


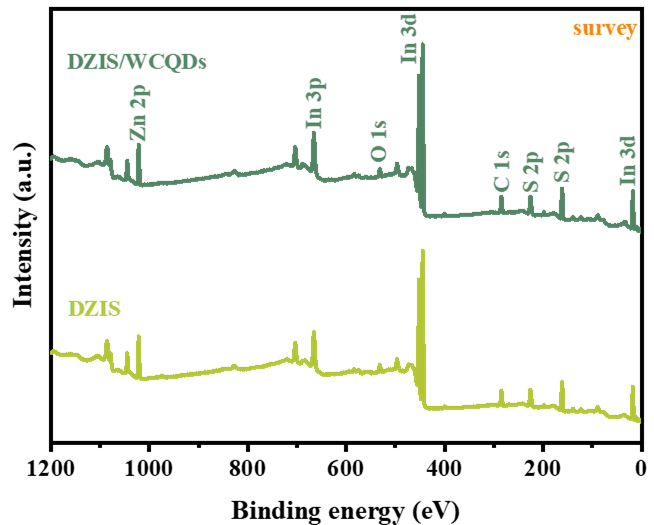


**Figure S6.** XPS spectra of full survey of DZIS and DZIS/WCQDs.


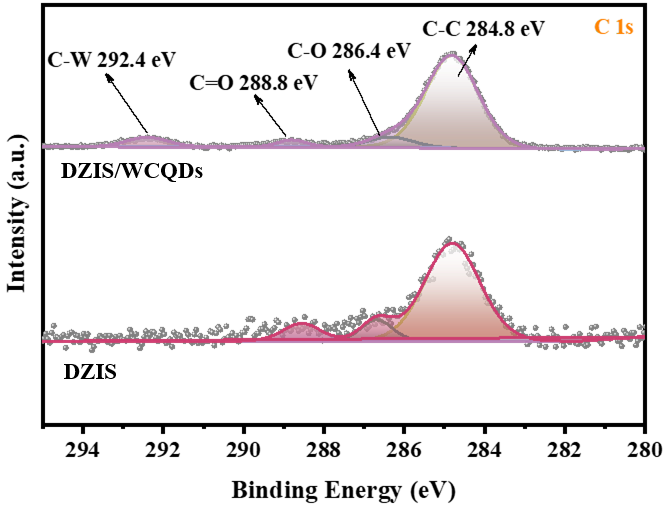


**Figure S7.** C 1s XPS spectra of DZIS and DZIS/WCQDs.


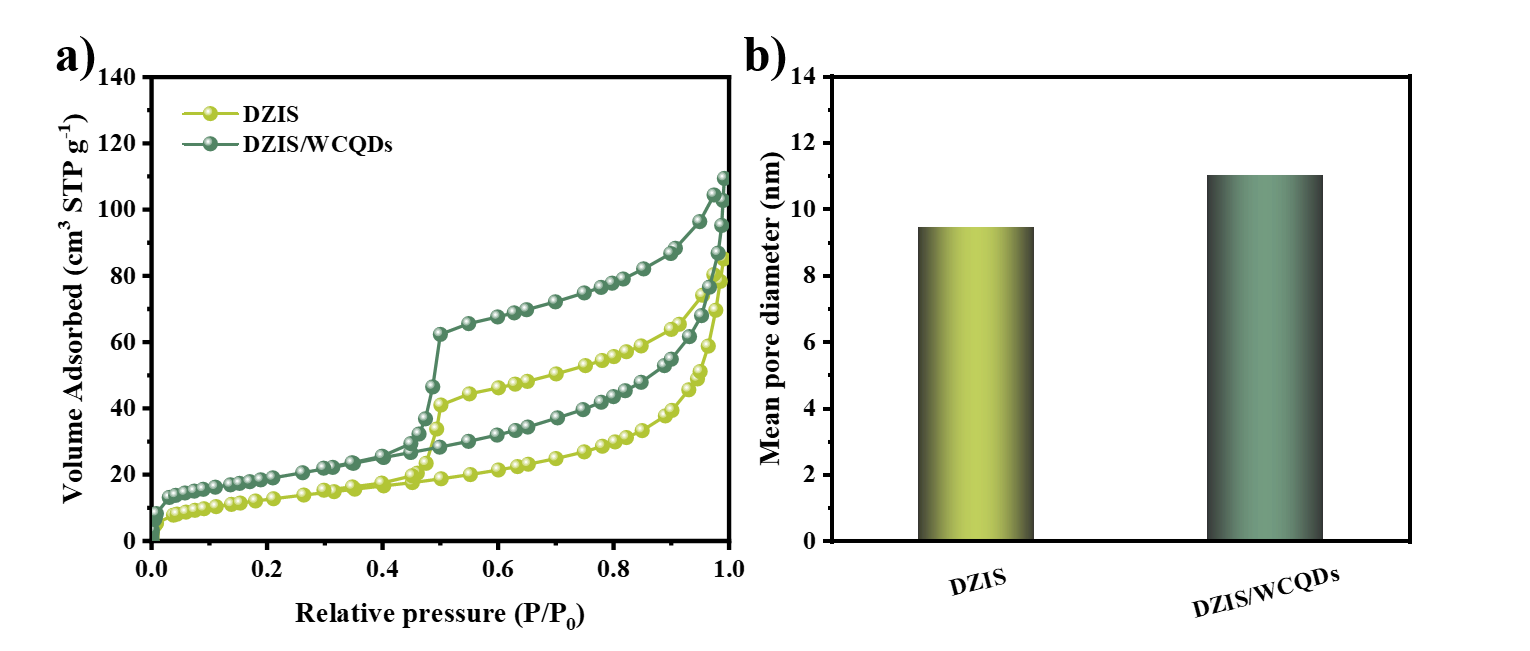


**Figure S8.** a) Nitrogen sorption isotherms and b) Mean pore diameter of DZIS and DZIS/WCQDs.

.


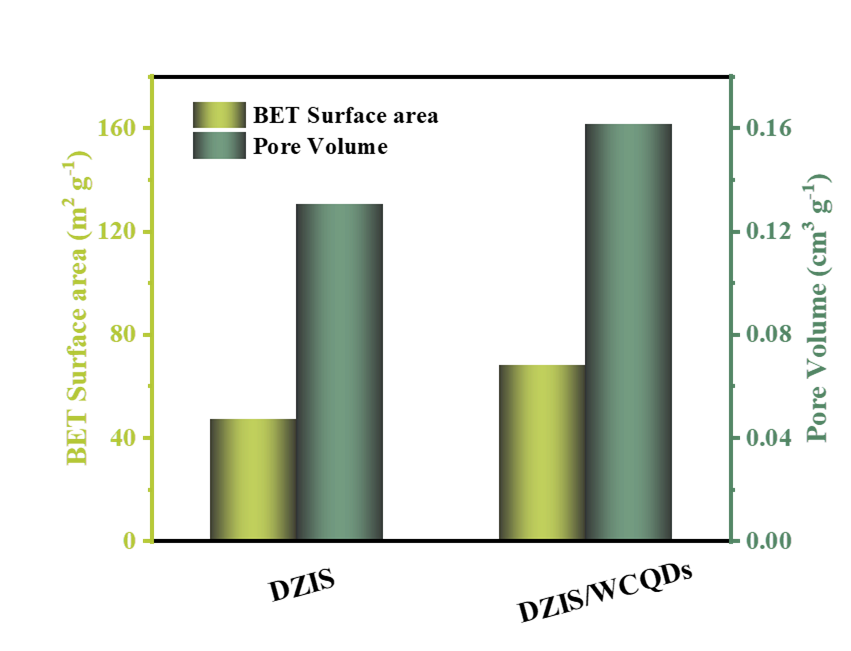


**Figure S9.** BET surface areas and pore volumes of DZIS and DZIS/WCQDs


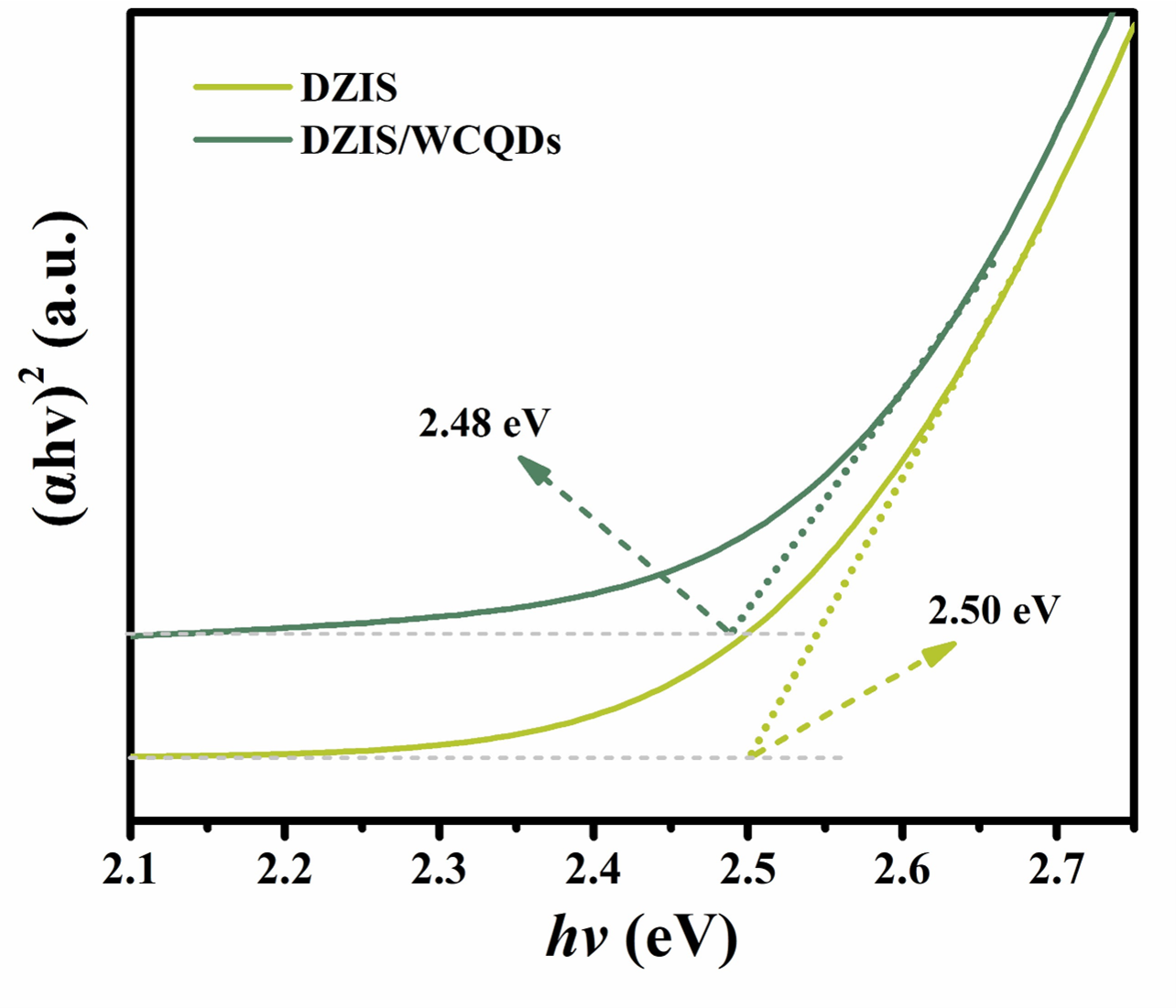


**Figure S10.** Optical bandgaps of DZIS and DZIS/WCQDs.


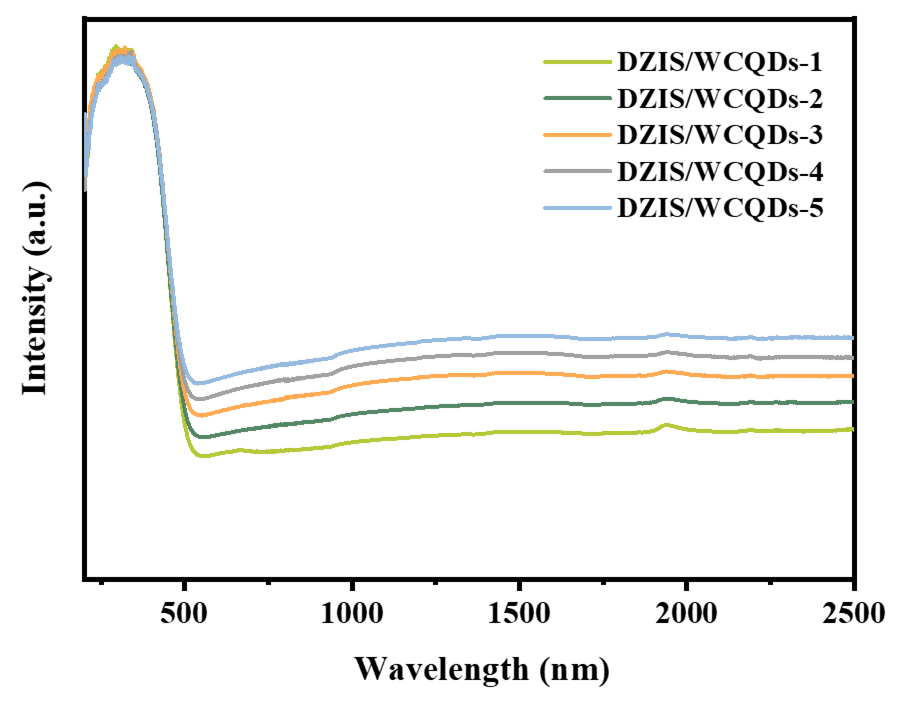


**Figure S11.** Ultraviolet-visible absorption spectra of DZIS/WCQDs with different WC QDs content.


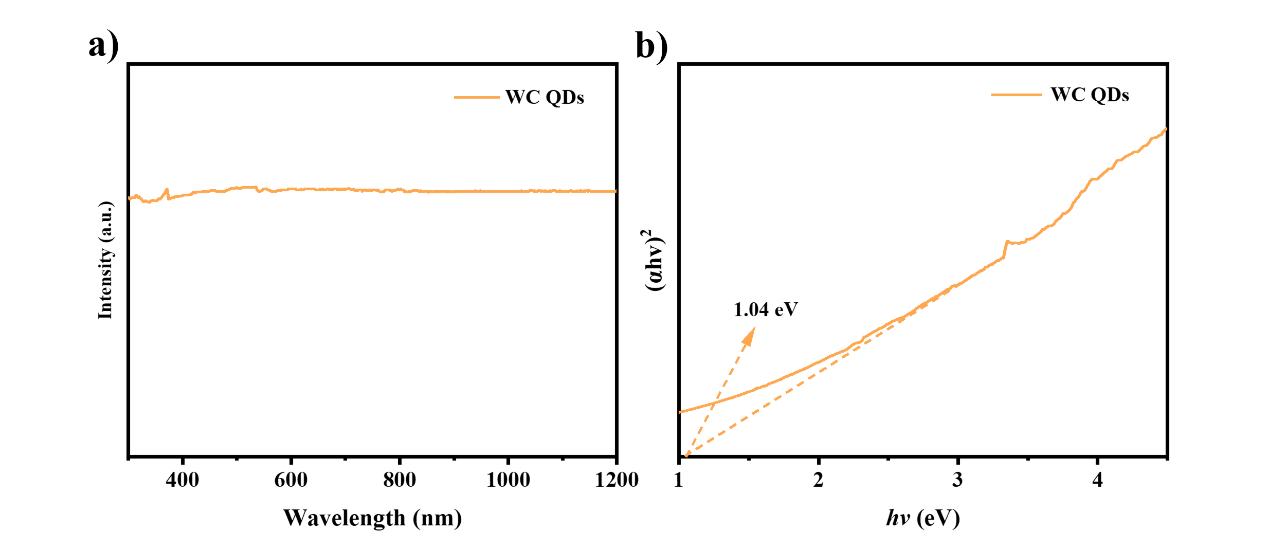


**Figure S12.** a) UV-vis absorption spectra and b) bandgaps of WC QDs.


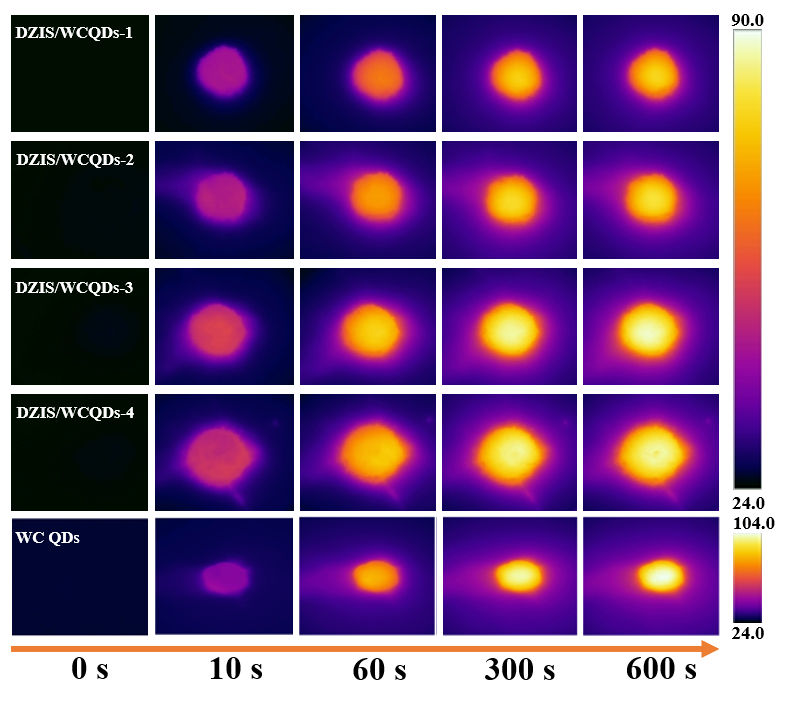


**Figure S13.** DZIS/WCQDs-n and WC QDs infrared thermal imaging images simulating sunlight exposure at different time points.


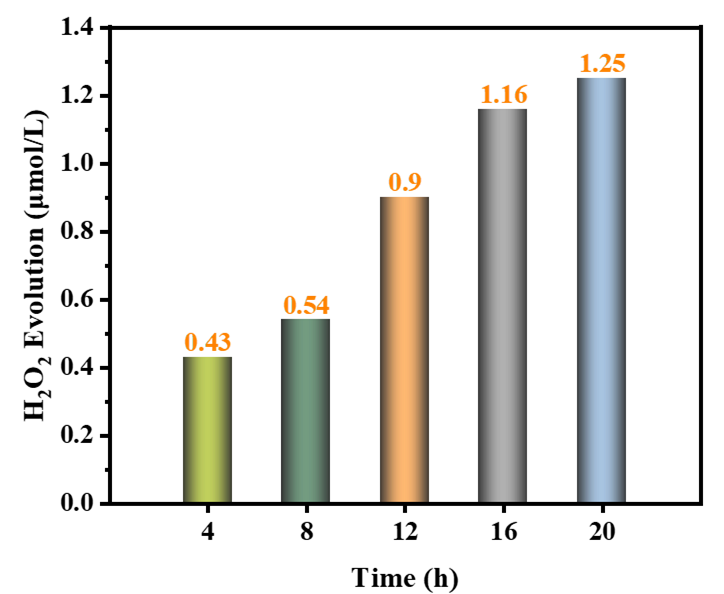


**Figure S14.** Time course of H_2_O_2_ concentration for DZIS/WCQDs (20 mg catalyst) in ultrapure water splitting without any sacrifice agent.


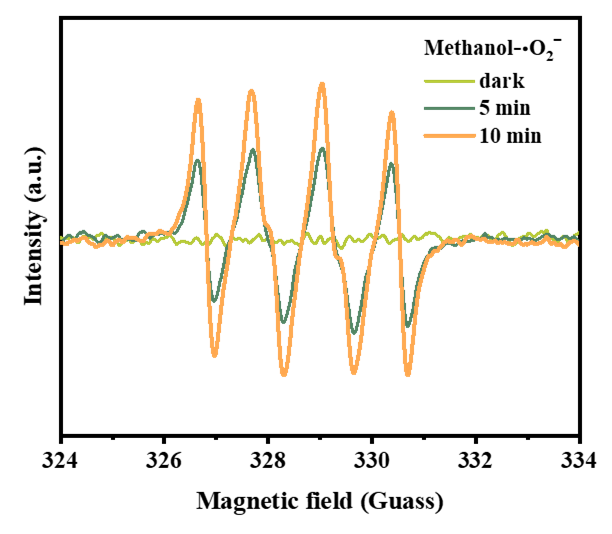


**Figure S15.** ESR detection of formed superoxide radical (•O_2_^-^) for DZIS and DZIS/WCQDs in methanol.

**
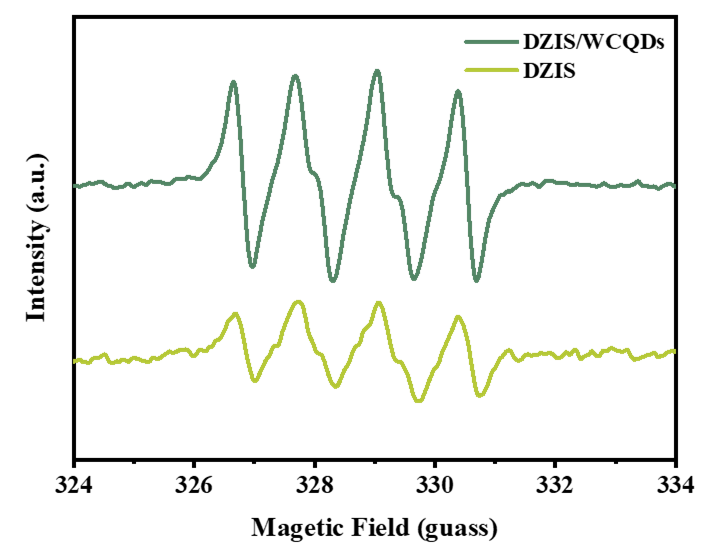
**

**Figure S16.** ESR detection of in-situ superoxide radical (•O_2_^-^) for DZIS/WCQDs with different light time conditions.

**
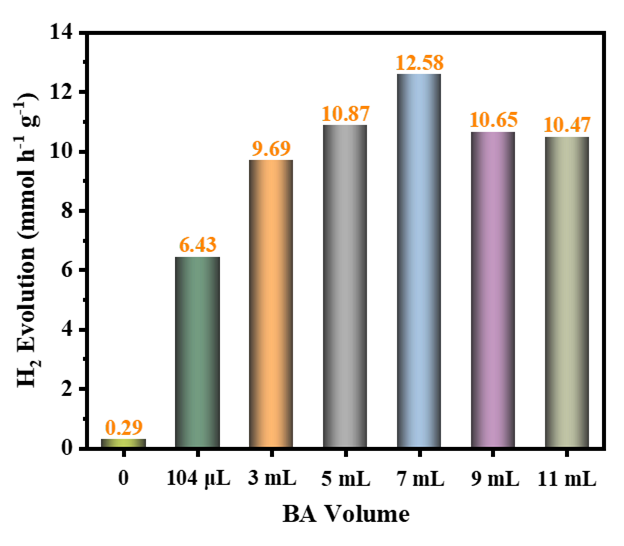
**

**Figure S17.** Effects of different volumes of BA on photocatalytic performance toward the H_2_ evolution.


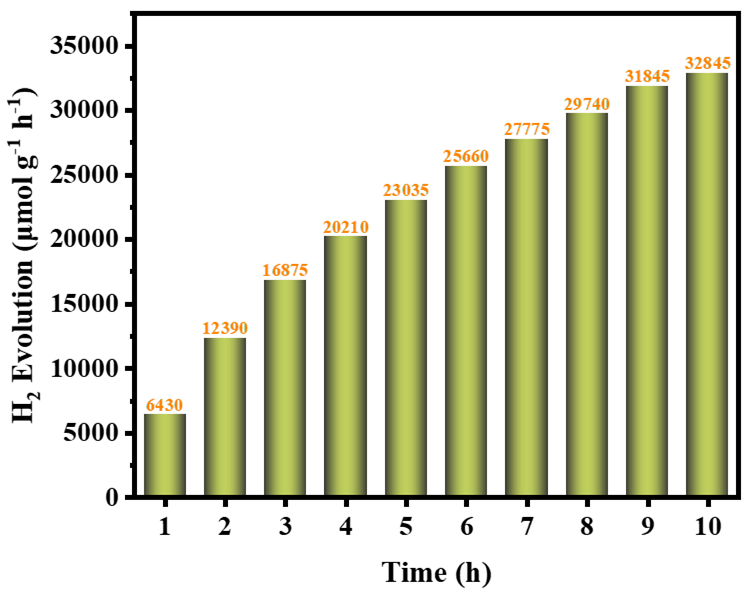


**Figure S18.** Histogram of hydrogen evolution of the coupling reaction between water-splitting and phenylcarbinol oxidation under time-extended solar irradiation of DZIS/WCQDs.


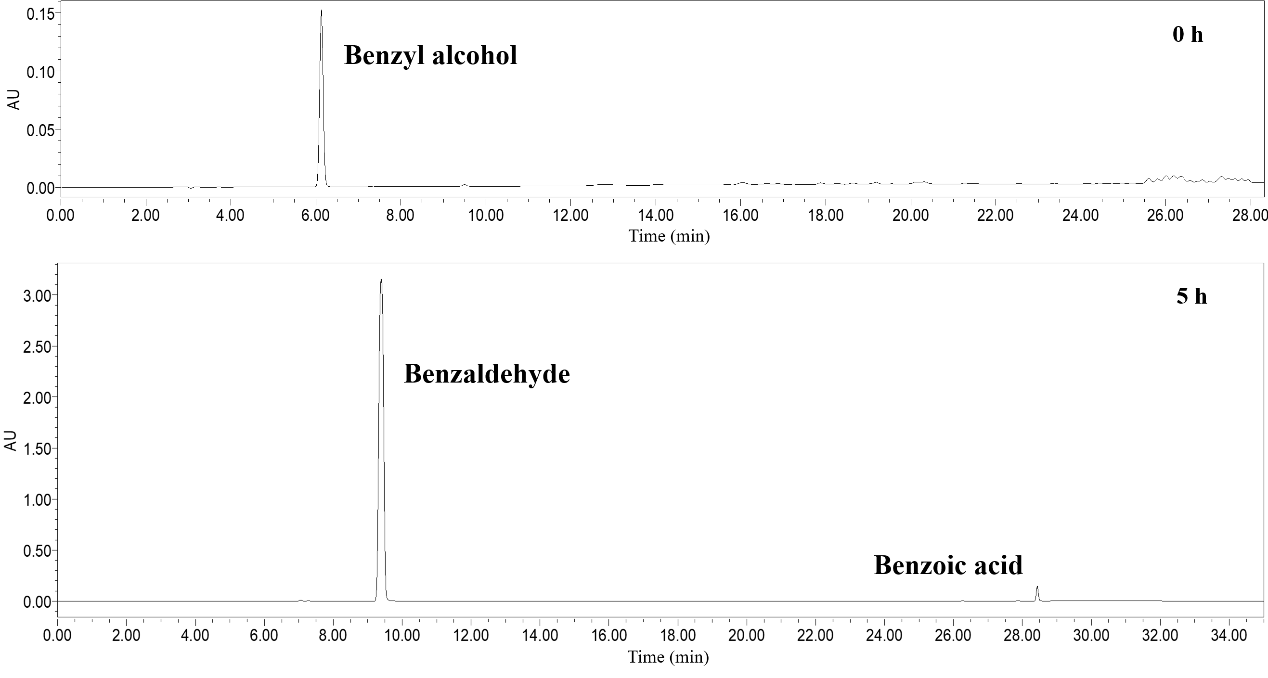


**Figure S19.** The high-performance liquid chromatograms before and after the photocatalytic reaction.


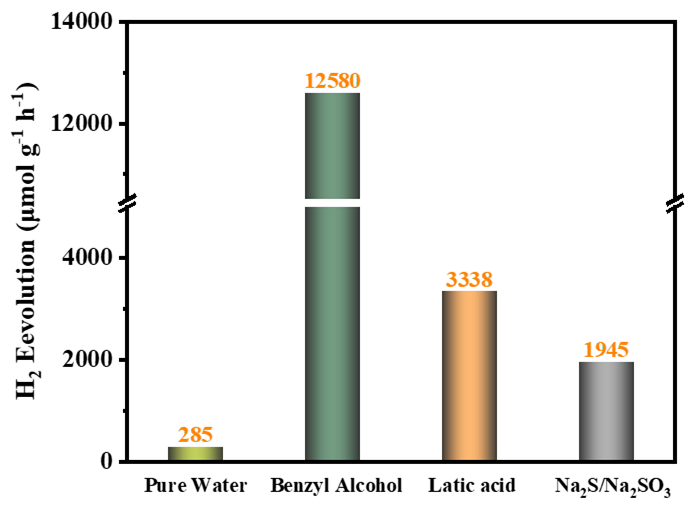


**Figure S20.** DZIS/WCQDs comparison of the photocatalytic hydrogen evolution properties of BA (104 μL）and pure water in different sacrificial agents.


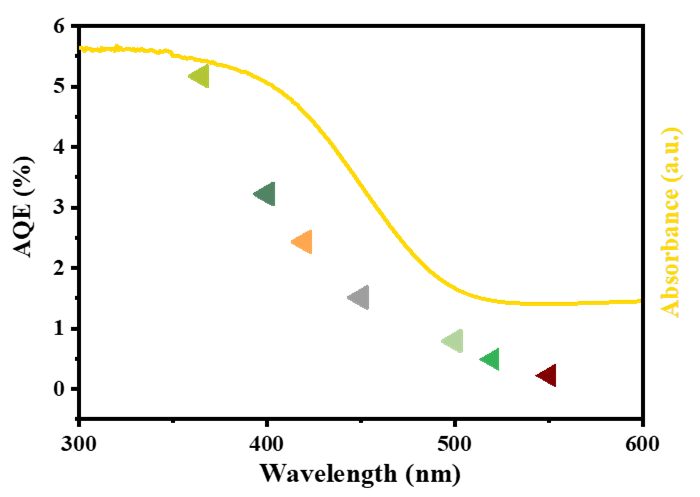


**Figure S21.** Wavelength-dependent AQE of DZIS/WCQDs.

**
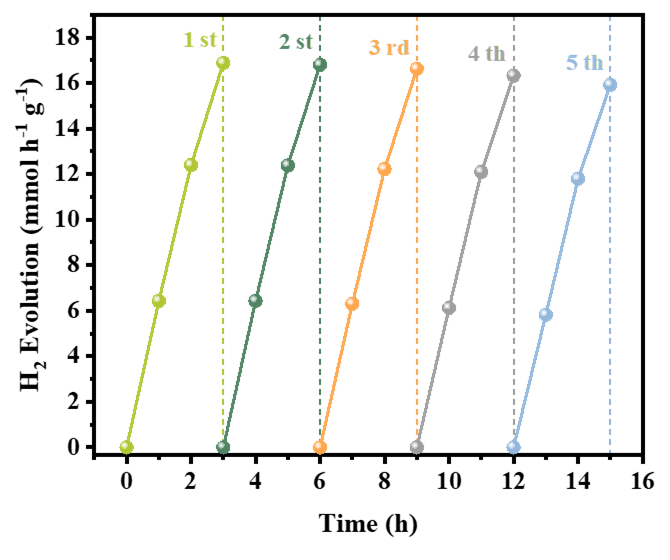
**

**Figure S22.** Photocatalytic cycling tests of DZIS/WCQDs in the BA–water system.


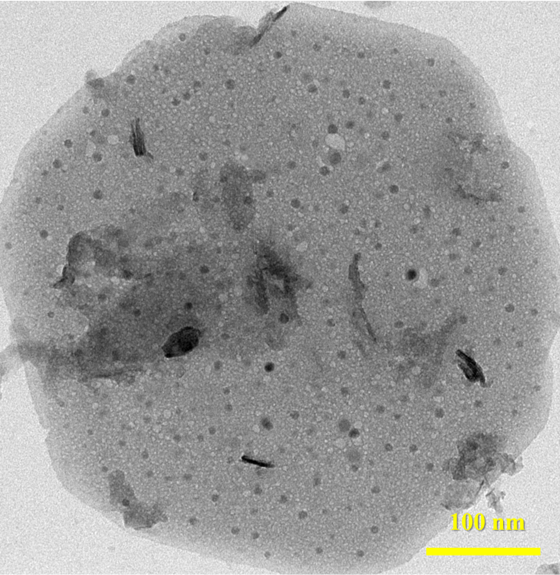


**Figure S23.** TEM spectra of the used DZIS/WCQDs composite**.**


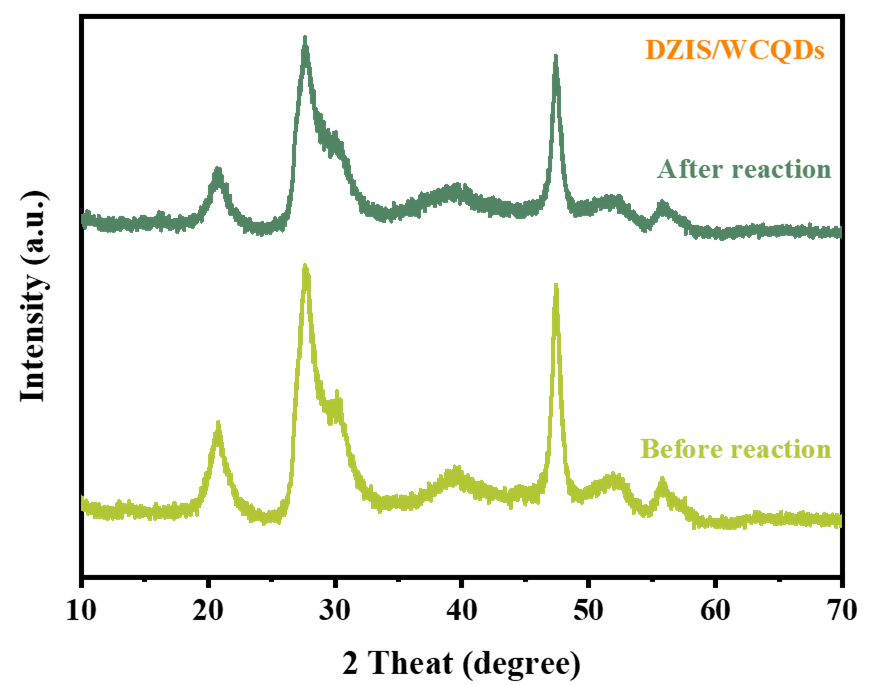


**Figure S24.** XRD spectra of DZIS/WCQDs composites before and after photocatalytic reaction.


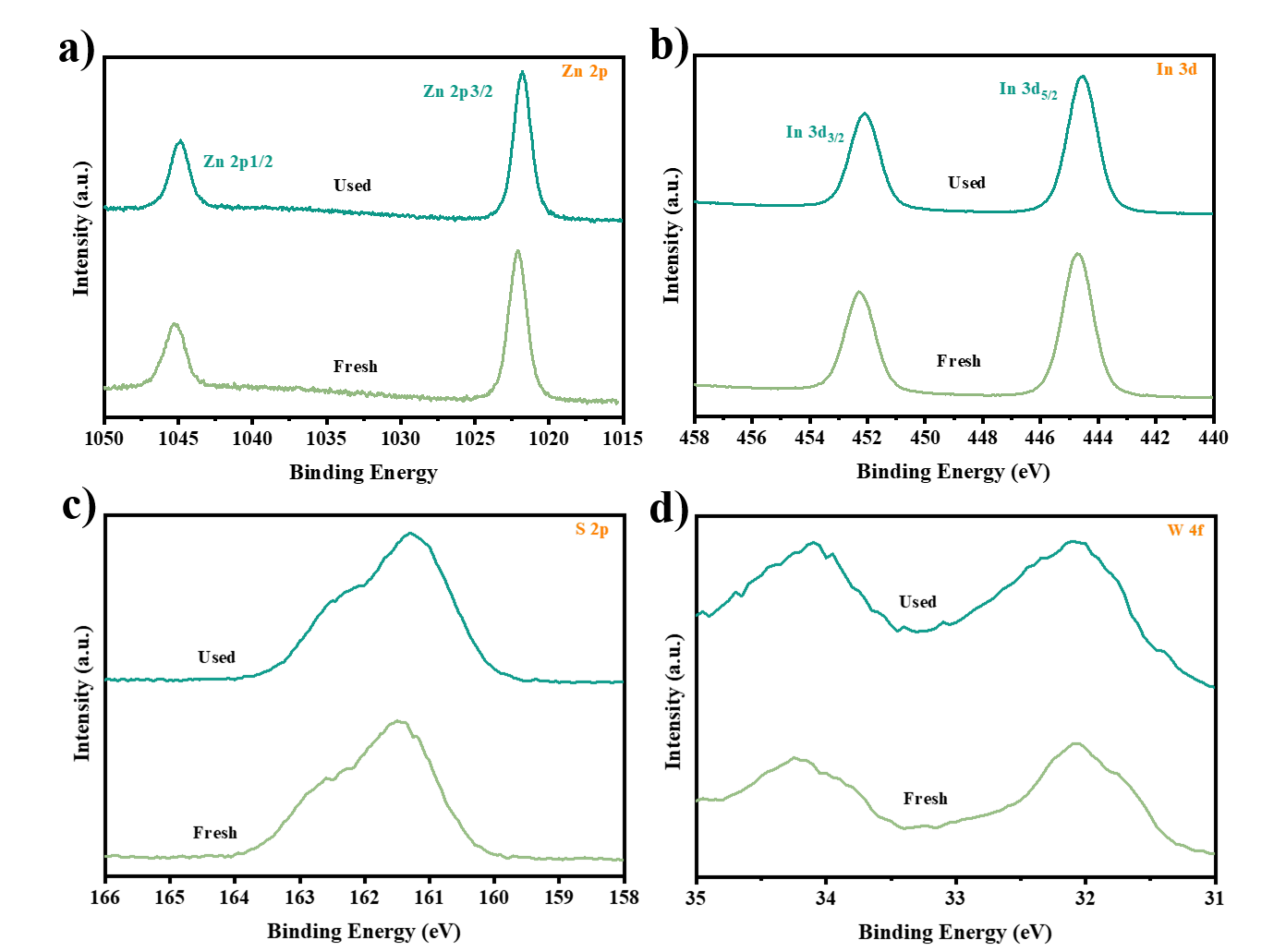


**Figure S25.** XPS spectra of DZIS/WCQDs composites before and after reaction.

**
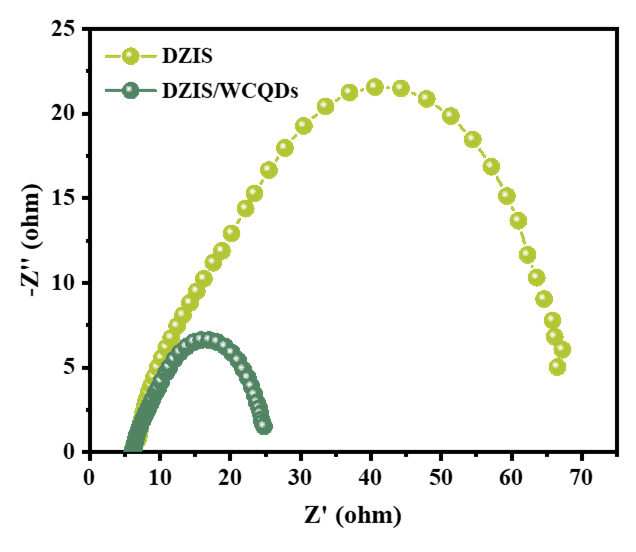
**

**Figure S26.** EIS Nyquist plots of DZIS and DZIS/WCQDs.


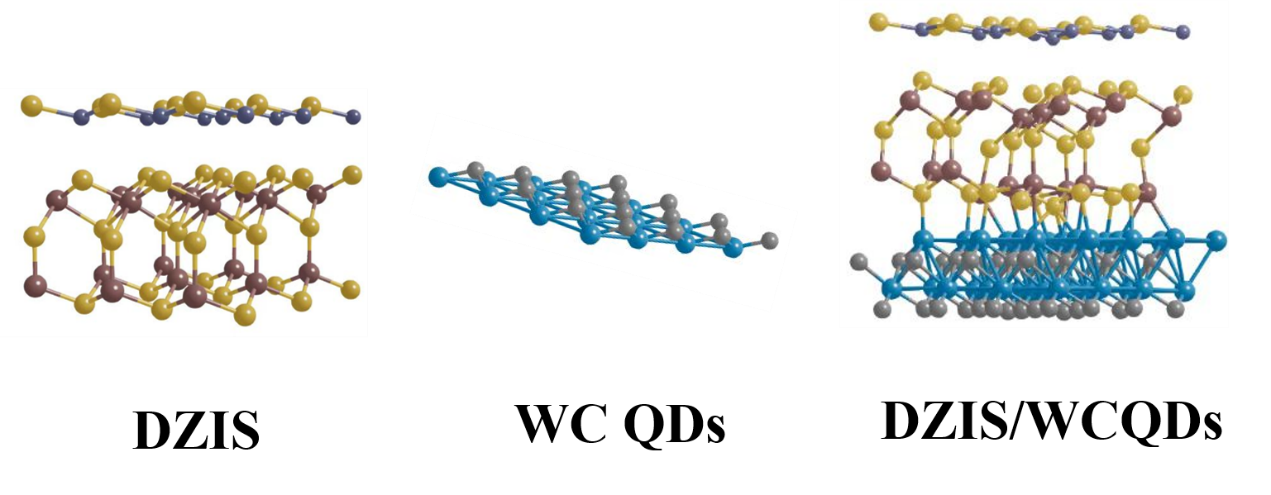


**Figure S27.** Construction model of DZIS, WC QDs and DZIS/WCQDs.


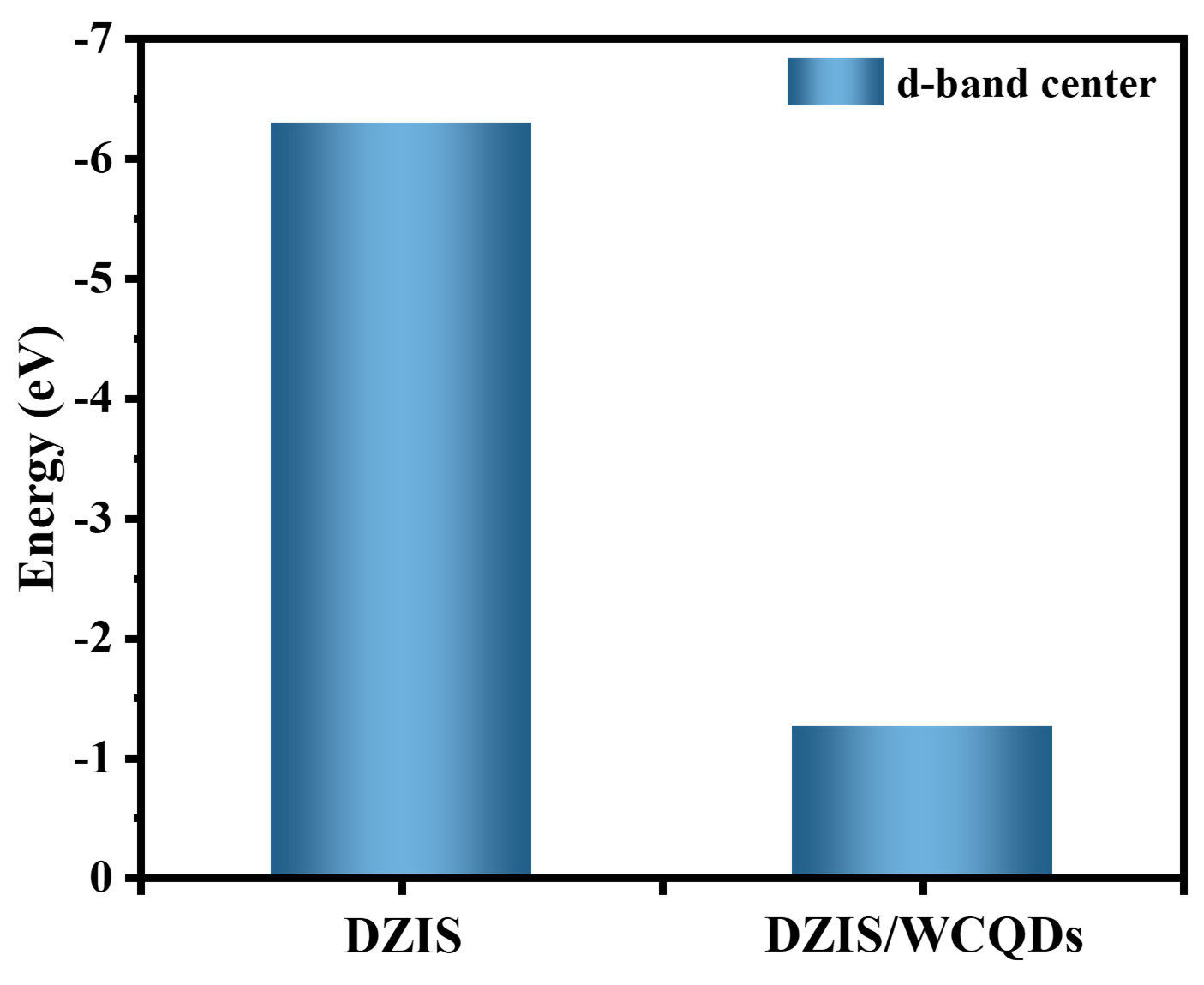


**Figure S28.** The D-band center value of DZIS and DZIS/WCQDs.


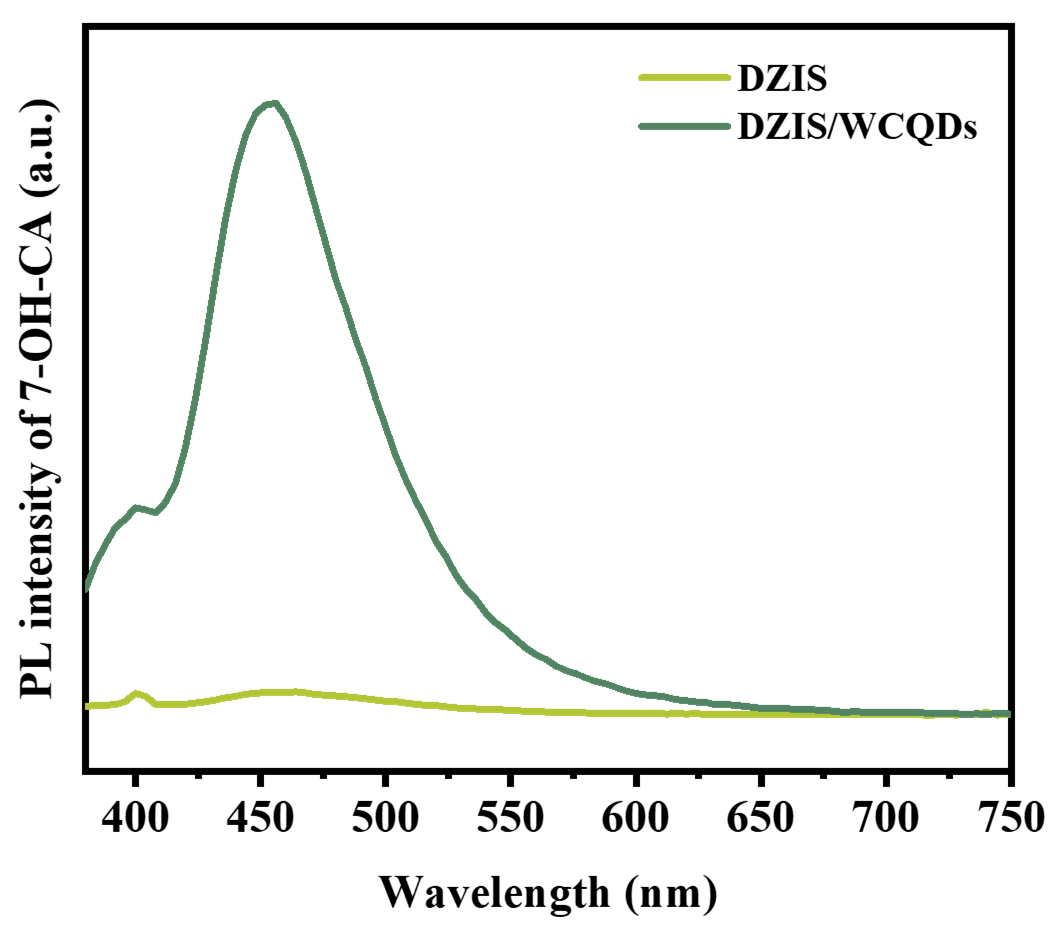


**Figure S29.** Comparison of hydroxyl radical capture of DZIS and DZISWCQDs.


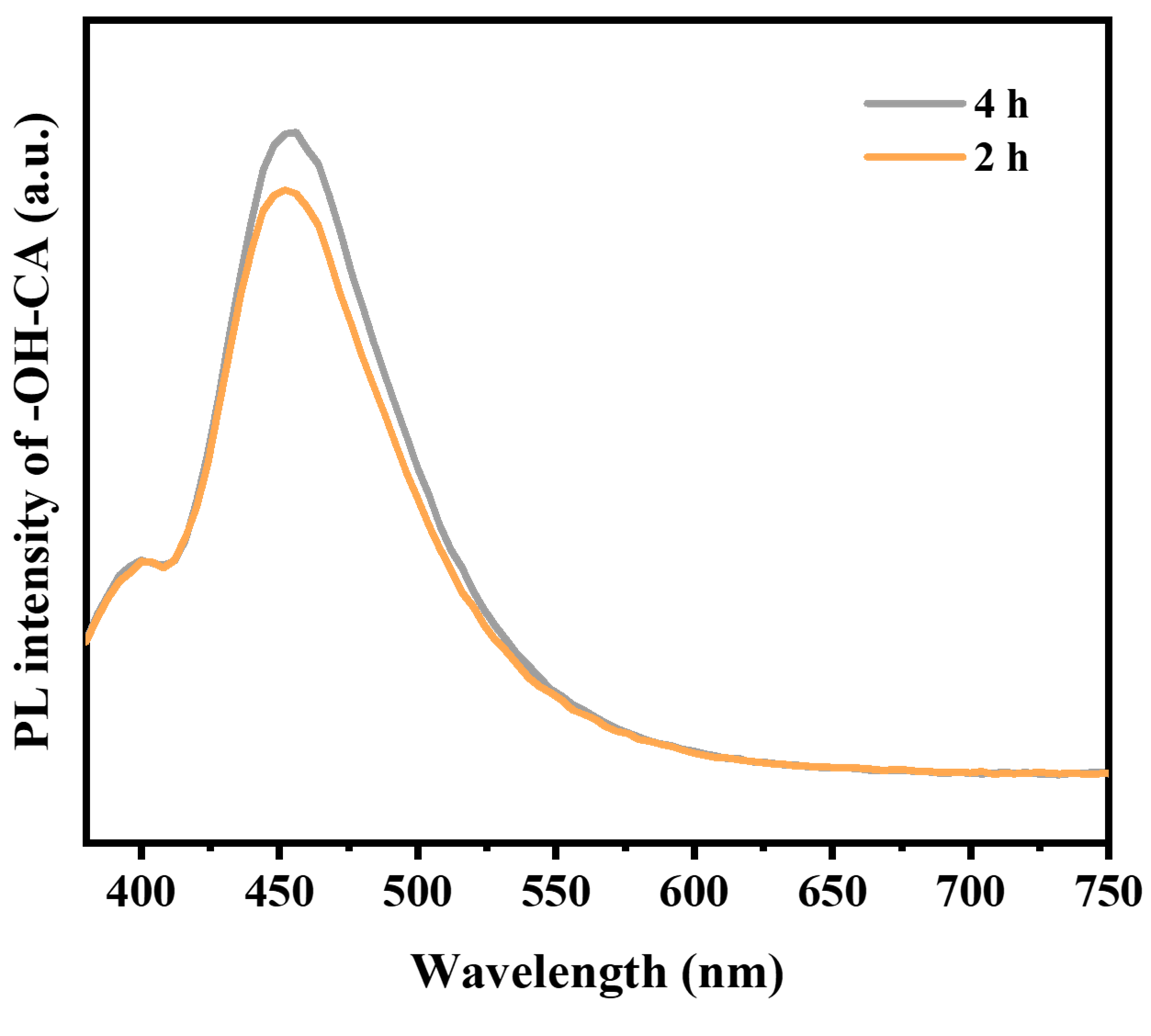


**Figure S30.** Comparison of hydroxyl radical capture of DZISWCQDs with different time.


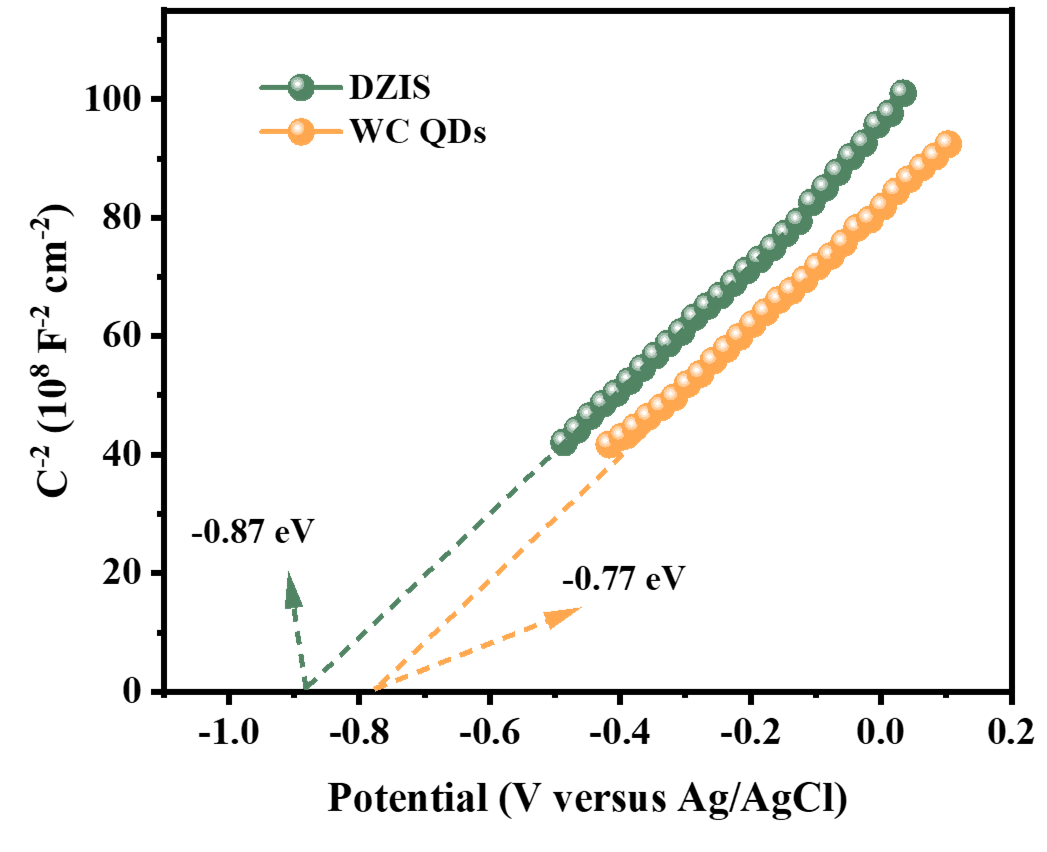


**Figure S31.** Mott-Schotty plots of DZIS and WCQDs.


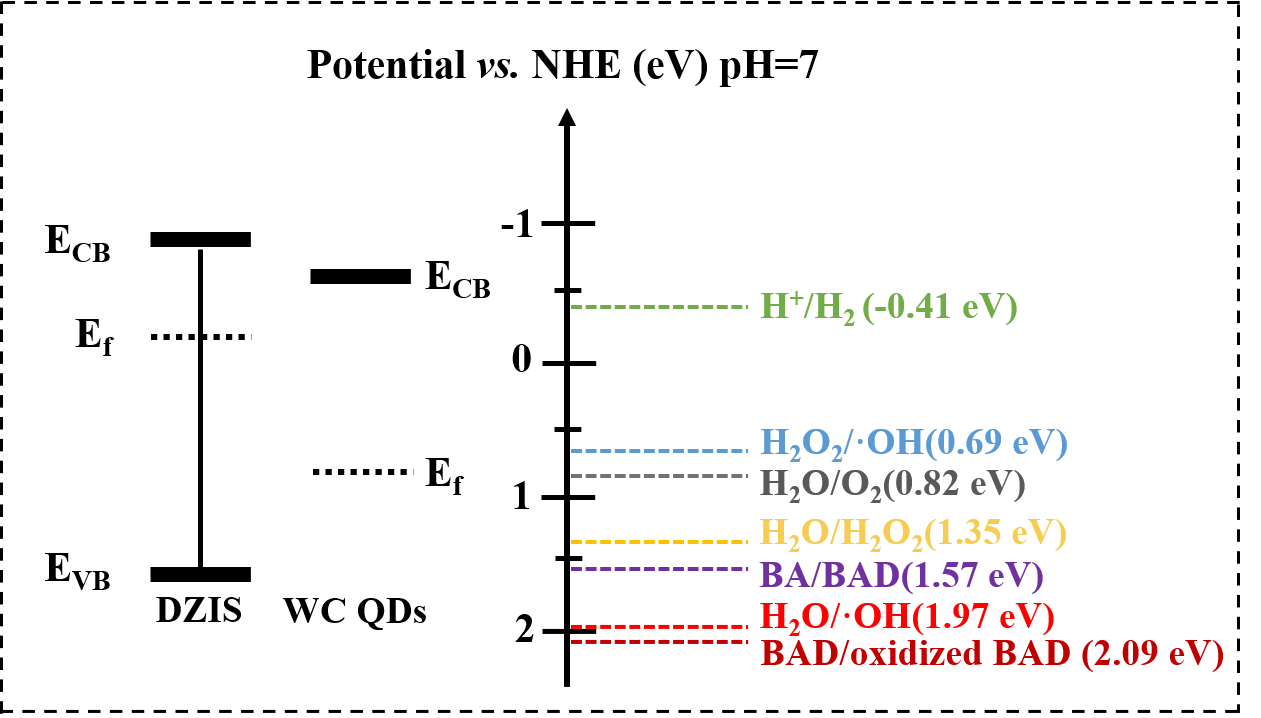


**Figure S32.** Conduction band and Fermi level positions of DZIS and WC QDs.

**Table S1.** Specific surface area and pore size of DZIS and DZIS/WCQDs samples.

| Sample | Specific area  (m^2^ g^-1^) | Mean pore diameter  (nm) | Total pore volume  (cm^3^ g^-1^) |
| --- | --- | --- | --- |
| DZIS | 47.35 | 9.45 | 0.13 |
| DZIS/WCQDs | 68.28 | 11.01 | 0.16 |

**Table S2.** Parameters obtained from time-resolved PL decay curves of DZIS and DZIS/WCQDs.

| Sample | τ_1_ (ns) | τ_2_ (ns) | A_1_ (%) | A_2_ (%) | τ_av_ (ns) |
| --- | --- | --- | --- | --- | --- |
| DZIS | 0.89 | 6.11 | 54.62 | 45.38 | 5.33 |
| DZIS/WCQDs | 0.66 | 8.94 | 39.56 | 66.44 | 8.56 |

**Table S3.** Performance comparison of recent research for selective oxidation of benzyl alcohols to benzaldehyde under anaerobic and aerobic conditions (aqueous medium), respectively.

| **Photocatalyst** | **Light source** | **Time (h)** | **Conv. (%)** | **Sel. of BAD (%)** | **BAD rate (mmol/g/h)** | **HER rate (mmol/g/h)** | **Ref.** |
| --- | --- | --- | --- | --- | --- | --- | --- |
| NiS/CdS | λ＞400 nm | 4 | 79 | 91 | 17.5 | 18.7 | S[1] |
| Co_2_P/Cd_0.9_Zn_0.1_S | λ＞420 nm | - | - | 92 | 16.5 | 15 | S[2] |
| CdS(ZB)/CdS(WZ)/Ni-BTC | λ＞420 nm | 3 | 99.0 | 90.2 | - | 2.891 | S[3] |
| ZnCo_2_S_4_/ Zn_0.2_Cd_0.8_S | λ＞400 nm | 18 | 54.3 | 92.2 | - | 23.02 | S[4] |
| WO_x_/CdS | λ＞420 nm | 7 | 91 | >99 | - | 0.13 | S[5] |
| POM/ZnIn_2_S_4_ | λ＞420 nm | 5 | 96.1 | 98.2 | - | 10.6 | S[6] |
| Ru/g-C_3_N_4-X_ | 320＜λ＜850 nm | 3 | - | >99 | 5.07 | 6.42 | S[7] |
| PSO-Fe_2_O_3_ | λ＞420 nm | 4 | - | - | 4.6 | 5.9 | S[8] |
| Ni/Cd_0.7_Mn_0.3_S | λ＞420 nm | 4 | 77 | 99 | 2.88 | 2.94 | S[9] |
| RuSA-RuO_2_/TiO_2_ | AM 1.5G | - | - | 99 | 1.42 | 2.91 | S[10] |
| Ni_x_-Zn_3_In_2_S_6_ | λ＞420 nm | 2 | - | 48.7 | - | 9.13 | S[11] |
| VC/CdS | λ＞420 nm | 2 | 41% | >99 | - | 20.5 | S[12] |
| Ni/ZnIn_2_S_4_ | λ＞420 nm | 5 | 97 | 92 | 3.608 | 1.46 | S[13] |
| CdS-Co(OH)_2_-GR | λ＞420 nm | 2 | 71 | 96 | - | 3.18 | S[14] |
| Mo-ZnIn_2_S_4_@Ti | - | 6 | - | >99 | - | 12 | S[15] |
| MoS_2_/ZnIn_2_S_4_ | λ＞420 nm | 5 | - | - | 3.69 | 3.88 | S[16] |
| Ni/(Au@CdS) | λ＞420 nm | - | - | >99 | 4.24 | 3.882 | S[17] |
| Pt-g-C_3_N_4_ | λ＞400 nm | 20 | 40 | 90 | - | 0.255 | S[18] |
| ZnS-Ni_x_S_y_ | λ＞200 nm | 3 | 42.1 | 90.5 | - | 2.943 | S[19] |
| CdS@MoS_2_ | λ＞420 nm | 3 | 42 | 99 | - | 4.23 | S[20] |
| WO_3_/ ZnIn_2_S_4_ | λ＞400 nm | 5 | 91.9 | 94.8 | - | 0.218 | S[21] |
| DZIS/WCQDs | AM 1.5G | 5 | 85.3 | 96.5 | 10.53 | 12.58 | This work |

**3. References**

[1] J. Luo, M. Wang, L. Chen, J. Shi, *J. Energy Chem.* **2022**, *66*, 52.

[2] Y. Yang, W. Ren, Y. Liu, C. Cai, X. Zheng, S. Meng, L. Zhang, *J. Colloid Interface Sci*. **2023**, *649*, 547.

[3] Y. Zhang, Z. Liu, C. Guo, T. Chen, C. Guo, Y. Lu, J. Wang, *Appl. Surf. Sci*, **2022**, *571*, 151284.

[4] C. Li, S. Shan, K. Ren, W. Dou, C. He, P. Fang. **2022**, *47*, 38951.

[5] J. Tian, X. Zhang, Z. Zhang, X. Ye, T. Zhang, W. Zeng, X. Guan, L. Guo, *Int. J. Hydrogen Energy*. **2024**, *74*, 31.

[6] F. Xing, R. Zeng, C. Cheng, Q. Liu, C. Huang, *Appl. Catal*. **2022**, *306*, 121087.

[7] Q. Yang, T. Wang, Z. Zheng, B. Xing, C. Li, B. Li, *Appl. Catal*. **2022**, *315*, 121575.

[8] J. Wu, Y. Wang, S. Zhang, Y. Liu, F. Wang, *Appl. Catal*, **2023**. *332*, 122741.

[9] H. Wen, W. Duan, L. Guo, Q. Wang, X. Fu, Y. Wang, R. Li, B. Jin, R. Du, C. Yang, D. Wang, *Appl. Catal*. **2024**, *345*, 123641.

[10] B. Xing, T. Wang, Z. Zheng, S. Liu, J. Mao, C. Li, B. Li, *Chem. Eng. J*. **2023**, *461*, 141871.

[11] D. Gunawan, L.Y. Lau, J.A. Yuwono, P.V. Kumar, L. Oppong-Antwi, I. Kuschnerus, S.L.Y. Chang, R.K. Hocking, R. Amal, J. Scott, C.Y. Toe, *Chem. Eng. J*. **2024**, *486*, 150215.

[12] M. Tayyab, Y. Liu, S. Min, R. Muhammad Irfan, Q. Zhu, L. Zhou, J. Lei, J. Zhang, *Chinese J. Catal*. **2022**, *43*, 1165.

[13] Q. Lin, Y.-H. Li, M.-Y. Qi, J.-Y. Li, Z.-R. Tang, M. Anpo, Y.M.A. Yamada, Y.-J. Xu, *Appl. Catal*. **2020**, *271*, 118946.

[14] Z.-G. Liu, Y. Wei, L. Xie, H.-Q. Chen, J. Wang, K. Yang, L.-X. Zou, T. Deng, K.-Q. Lu, *Mol. Cata*l. **2024**, *553*, 113738.

[15] J. Li, C. Guo, Y. Niu, X. Cao, J. Li, J. Wang, *Inorg. Chem*. **2024**, *63*, 9297.

[16] Z.-H. Chen, Y.-H. Li, M.-Y. Qi, Z.-R. Tang, Y.-J. Xu, *Res. Chem. Intermed*. **2022**, *48*, 1.

[17] Z. Zheng, T. Wang, F. Han, Q. Yang, B. Li, *J. Colloid Interface Sci*. **2022**, *606*, 47.

[18] F. Li, Y. Wang, J. Du, Y. Zhu, C. Xu, L. Sun, *Appl. Catal*. **2018**, *225*, 258.

[19] H. Hao, L. Zhang, W. Wang, S. Qiao, X. Liu, *ACS Sustainable Chem Eng.* **2019**, *7*, 10501.

[20] P. Li, H. Zhao, X. Yan, X. Yang, J. Li, S. Gao, R. Cao, *Sci. China Mater.* **2020**, *63*, 2239.

[21] Y.-H. Li, M.-Y. Qi, Z.-R. Tang, Y.-J. Xu, *J. Phys. Chem. C.* **2022**, *126*, 1872.
